# The high-throughput solid-phase extraction of *cis*-cyclo(L-Leu-L-Pro) and *cis*-cyclo(L-Phe-L-Pro) from *Lactobacillus plantarum* demonstrates efficacy against multidrug-resistant bacteria and influenza A (H3N2) virus

**DOI:** 10.3389/fmolb.2024.1346598

**Published:** 2024-05-17

**Authors:** Jaeyoung Son, Yeonju Hong, Hyeri Seong, Yoon Sin Oh, Min-Kyu Kwak

**Affiliations:** ^1^ Laboratory of Microbial Physiology and Biotechnology, Department of Food and Nutrition, Institute of Food and Nutrition Science, College of Bio-Convergence, Eulji University, Seongnam, Republic of Korea; ^2^ Department of Food and Nutrition, Institute of Food and Nutrition Science, College of Bio-Convergence, Eulji University, Seongnam, Republic of Korea

**Keywords:** cyclic dipeptides, *cis*-cyclo(L-Leu-L-Pro), *cis*-cyclo(L-Phe-L-Pro), solid-phase extraction, influenza A virus, *Lactobacillus* plantarum LBP-K10

## Abstract

**Introduction:**

2,5-diketopiperazines are the simplest forms of cyclic dipeptides (CDPs) and have diverse frameworks with chiral side chains that are useful for drug development. Previous research has investigated the antimicrobial properties of proline-linked CDPs and their combinations in the culture filtrate (CF) of *Lactobacillus plantarum* LBP-K10 using anion exchange chromatography (AEC). However, the quantity of CDPs showcasing notable anti-influenza virus activity derived from AECs was generally lower than those originating from *Lactobacillus* CF.

**Methods:**

To address this issue, the study aims to propose a more efficient method for isolating CDPs and to introduce the antiviral combinations of CDPs obtained using a new method. The study employed a novel technique entailing high-throughput C18-based solid-phase extraction with a methanol gradient (MeSPE). The MeSPE method involved increasing the methanol concentration from 5% to 50% in 5% increments.

**Results:**

The methanol SPE fractions (MeSPEfs) eluted with methanol concentrations between 35% and 45% evinced substantial efficacy in inhibiting the influenza A/H3N2 virus via plaque-forming assay. MeSPEf-45, the 45% MeSPEf, exhibited exceptional efficacy in preventing viral infections in Madin-Darby kidney cells, surpassing both individual CDPs and the entire set of MeSPEfs. To identify the specific antiviral components of MeSPEf-45, all MeSPEfs were further fractionated through preparative high-performance liquid chromatography (prep-HPLC). MeSPEf-45 fractions S8 and S11 presented the highest activity against multidrug-resistant bacteria and influenza A/H3N2 virus among all MeSPEfs, with 11 common fractions. Antiviral fractions S8 and S11 were identified as proline-based CDPs, specifically *cis*-cyclo(L-Leu-L-Pro) and *cis*-cyclo(L-Phe-L-Pro), using gas chromatography-mass spectrometry. The combination of MeSPEf-45 fractions S8 and S11 displayed superior antibacterial and anti-influenza virus effects compared to the individual fractions S8 and S11.

**Discussion:**

High-throughput MeSPE-derived MeSPEfs and subsequent HPLC-fractionated fractions presents an innovative approach to selectively purify large amounts of potent antimicrobial CDPs from bacterial CF. The findings also show the effectiveness of physiologically bioactive combinations that utilize fractions not containing CDP. This study provides the initial evidence demonstrating the antimicrobial properties of CDPs acquired through high-throughput SPE techniques.

## Introduction

Postbiotics, which are derived from microbiome research, consist of metabolic byproducts, non-viable microbial cells, and their components (Aggarwal et al., 2022). These postbiotics exhibit antimicrobial, antioxidant, and immunomodulatory activities, resulting in various physiological, immunological, neuro-hormonal, regulatory, and metabolic reactions (Ji et al., 2015; Cuevas-González et al., 2020). Postbiotics produced by microorganisms include physiologically active and antiviral substances found in the culture filtrates (CFs) of probiotic lactic acid bacteria (LAB) (AI Kassaa et al., 2014; Archer and Halami, 2015). The filtrates primarily consist of low-molecular-weight compounds, such as hydrogen peroxide (H_2_O_2_), carbon dioxide (CO_2_), organic acids (RCOOH, RSOOOH, ROH), diacetyl [(CH₃CO)₂)], acetaldehyde (CH₃CHO) (Rouse and van Sinderen, 2008; Crowley et al., 2013). The CFs contain proteinaceous or peptidic toxins known as bacteriocins, which are the most abundant and diverse group of bacterial defense systems. Probiotics and their prebiotics have a broad spectrum of antimicrobial and disease therapeutic potential (Özogul and Hamed, 2018; Sharma et al., 2021). Additionally, 2,5-diketopiperazines (2,5-DKPs), the simplest form of cyclic dipeptides (CDPs), offer a promising avenue for drug development. CDPs have the potential to serve as reservoirs for medically significant antiviral compounds due to their distinctive orbital and target-specific chirality, diverse structural nature, and potential application of cyclotides and relevant peptide scaffolds for creating novel bioactive molecules based on DKPs in the pharmaceutical sector (Borthwick, 2012; Bellezza et al., 2014). CDP derivatives, obtained from natural sources or chemical synthesis, are essential active compounds with diverse biological functions. These properties include bacterial quorum sensing, antibacterial, antimicrobial, and anticancer effects, neuroprotective functions, and serving as carriers for essential molecules through the blood-brain barrier (Martins and Carvalho, 2007). CDPs offer simple yet effective structures for self-assembly, enabling molecular recognition, water solubility, thermal stability, biocompatibility, and versatile structures for developing new therapeutic approaches with improved effectiveness and fewer side effects (Manchineella et al., 2017). A self-assembly is the process of linking molecules through noncovalent interactions to form functional systems known as supermolecules. It can be observed in living organisms, from bacteria to humans. The arrangement of bio-machinery, such as the DNA double helix, ribosomes, enzymes’ quaternary structure, and bio-entities like cell membranes, collagen’s helical structure, or cytoskeleton, is essential (Manchineella and Govindaraju, 2017). Non-covalent interactions at the supramolecular level create systems that aid in understanding bio-processes and developing innovative bio-materials. These materials can enhance 3D cell growth, proliferation, and differentiation (Chibh et al., 2021).

The significance of CDPs lies in their effectiveness as antimicrobial agents and their physiological implications for human diseases. Cyclo(His-Leu), originating from *Bacillus subtilis* B38, displays both antimicrobial and antioxidative properties (Elkahoui et al., 2013). Cyclo(L-Pro-D-Arg), produced by *Bacillus cereus*, a bacterium associated with a rhabditid entomopathogenic nematode, acts as a negative regulator of chitinase enzyme activity and has antibacterial and antitumor effects (Kumar et al., 2014). Cyclo(dehydroAla-Leu) and *cis*-cyclo(prolyl-valyl), synthesized by *Penicillium* sp. F70614 and *Pediococcus italicum* FUN2 respectively, exhibit inhibitory effects on α-glucosidase activity (Kwon et al., 2000; Nagia et al., 2012). High doses of cyclo(His-Pro) combined with zinc effectively lower hyperglycemia and blood glucose levels in both healthy human subjects and obese diabetic (ob/ob) mice (Uyemura et al., 2010; Jung et al., 2016). Cyclo(His-Phe) and cyclo(His-Tyr) demonstrate inhibitory effects on the growth of bacteria and fungi. These CDPs affect intracellular ion channels, leading to cell death in cervical (HeLa), esophageal (WHCO3), and breast (MCF-7) carcinoma cell lines (McCleland et al., 2004). Additionally, CDPs prove to be highly effective in treating a range of conditions, such as dermatitis, dementia, diabetes mellitus, pancreatic disorders, and neurodegenerative disorders (Tsuruoka et al., 2012; Song et al., 2018). Despite the extensive research on the effects of CDPs on pathogenic microorganisms and human diseases, there remains a lack of studies focusing on the underlying mechanisms that contribute to the antiviral properties of CDPs. However, the majority of studies on CDPs reveal antibacterial activity, with some suggesting antifungal effects. The prior research presents proof of the antimicrobial properties of CDPs, such as cyclo(dehydroAla-Leu), *cis*-cyclo(prolyl-valyl), cyclo(Pro-Trp), cyclo(Phe-Pro), cyclo(Trp-Pro), and cyclo(Trp-Trp) (Graz et al., 1999). These findings suggest that the potential of CDPs lies in their ability to inhibit the proliferation of virulent virus strains *in vitro* (Martins and Carvalho, 2007; Lee et al., 2018). Cyclo(Leu-Pro) from Goishi tea, along with proline-containing CDPs from *Lactobacillus plantarum* LBP-K10 display inhibitory effects against H1N1 and H3N2 influenza viruses (Kwak et al., 2013; Yoshioka, 2013). Cyclo(Phe-Tyr) and cyclo(Ala-Ile) derived from Moslae Herba exhibit antiviral activity against both the H1N1 and H3N2 strains of the influenza A virus (Zhang et al., 2018).

We previously reported two types of proline-containing CDPs produced by *Lb. plantarum* LBP-K10, namely, *cis*-cyclo(L-Leu-L-Pro) and *cis*-cyclo(L-Phe-L-Pro), which exhibit activity against influenza A (H3N2) virus (Kwak et al., 2013). We further studied the antimicrobial effects of various proline-based CDPs acquired via anion exchange chromatography (AEC) by utilizing Amberlite IRA-67 and Purolite A420S resins to remove organic acids and sugars from *Lb. plantarum* LBP-K10 CF. Even though AECs aid in investigating the combined synergistic activity of CDP mixtures against pathogenic microbes, their use in experiments presents several limitations and challenges. To improve the accuracy of our findings, we aimed to validate a new experimental technique that employs high-throughput solid-phase extraction (SPE) in combination with preparative high-performance liquid chromatography (prep-HPLC) fractionation.

In this study, we put forward a hypothesis regarding the potential production of CDPs dependent on biosynthesizing building blocks. These CDPs may play a role as biologically antiviral agents, particularly focusing on the yet undiscovered synergistic effect between small compounds 2,5-DKPs. These compounds are experimentally expected to be derived from the combination of LAB-fermented raw material CFs and methanol-based SPE techniques. Previous research has shown that antimicrobial CDPs containing proline, derived from bacterial CFs and naturally fermented plant-based foods, commonly exhibit antagonistic and antimicrobial activities (Kwak et al., 2018). Previous studies have investigated CDPs originating from both plants and animals. For example, CDPs sourced from plants like kimchi and CDPs from animals were discovered after enriching quail eggs with probiotics (Liu et al., 2017; Kang and Kwak, 2024). However, the experimental trials in this study differ significantly from our prior research in that they significantly reduce the loss of CDP yields through a newly used method. Through the functions of sample concentration and purification, SPE effectively circumvents these issues. Furthermore, SPE is highly compatible when combined with HPLC or HPLC-MS, making it the most effective and versatile method for sample pretreatment (Yan et al., 2008). Liquid chromatography (LC) or gas chromatography (GC) coupled with mass spectrometry (MS) and Tandem mass spectrometry (MS/MS) are currently the most extensively used methods to detect trace amounts of dipeptidyl molecules in water due to their high selectivity and sensitivity (Shimada et al., 2001). However, exact determination of trace analytes is impossible without sample preparation due to their extremely low concentration, which is usually lower than the instrumental limit of detection (LOD), and complex matrices that result in ionization suppression of electrospray ionization (ESI) (Laganà et al., 2000; Beck et al., 2008). A proper sorbent is crucial for SPE procedures to guarantee adequate recovery and extraction efficiency. The most frequently utilized sorbents in SPE are C18 bonded silica and a variety of polymeric materials. These sorbents are deemed suitable for the traditional analysis of trace analytes in low-concentration solutions. In the pursuit of high throughput and reduced solvent usage, there has been a surge of interest in the exploration and production of novel materials for application as sorbents in SPE methods. This trend has gained significant momentum in the past decade, signifying a vibrant area of research and development in SPE methodologies.

It is currently unknown whether any form of CDP or its derivatives can be detected through SPE experiments. Based on our hypothesis, this study aimed to investigate the effect of SPE resins on the yield of antiviral CDPs, due to the unique performance of SPE. The combination of C18-based SPE-derived fractions showed greater activity against influenza A virus compared to the CDP mixtures obtained from AEC. This technique provides a more efficient purification strategy for larger quantities of antiviral CDPs.

## Materials and methods

### Strains and culture condition

The bacterial and viral strains used in this study are listed (Table 1). The *Lb. plantarum* LBP-K10 strain was grown on either de Man, Rogosa and Sharpe (MRS) agar or broth, and consistently maintained in modified MRS (mMRS, MRS without beef extract) broth, as previously described (Kwak et al., 2018).

**TABLE 1:** The bacterial and viral strains employed in this study consist of Gram-positive and Gram-negative multidrug-resistant bacteria, bacterial indicator strains, and the influenza A/H3N2 virus.

Strain	Types or strains	Source or References
LAB strains		
* Lb. plantarum* LBP-K10	Original isolate from fermented Chinese cabbage	This study, (Kwak et al., 2018)
* Leuconostoc mesenteroides* LBP-K06	Original isolate from fermented Chinese cabbage	This study, (Kwak et al., 2018)
* Pediococcus pentosaceus* LBP-K17	Original isolate from fermented Chinese cabbage	This study, (Kwak et al., 2018)
^1^Virus		
* *Influenza A virus	H3N2	This study, KNIH (Kwak et al., 2018)
Multidrug-resistant bacteria^*a*^		
Gram-positive bacteria		
* S. aureus* 11,471	Oxacillin-resistant *S. aureus* (ORSA) 11,471, which is resistant to beta-lactam antibiotics, including penicillins (methicillin, dicloxacillin, nafcillin, and oxacillin) and cephalosporins	This study, KNIH (Kwak et al., 2018)
* *Gram-negative bacteria		
* S.* Typhimurium 12,219	*Salmonella* Typhimurium 12,219, which is resistant to ACSSuT (ampicillin, chloramphenicol, streptomycin, sulphonamides, and tetracycline)	This study, KNIH (Kwak et al., 2018)
^*2*^ Bacterial indicator strains		
Gram-positive bacteria		
	*Bacillus subtilis*	This study, KNIH (Kwak et al., 2018)
	*Staphylococcus aureus*	This study, KNIH (Kwak et al., 2018)
	*Streptococcus pneumoniae*	This study, KNIH (Kwak et al., 2018)
Gram-negative bacteria		
	*Salmonella* Typhimurium	This study, KNIH (Kwak et al., 2018)
	*Escherichia coli*	This study, KNIH (Kwak et al., 2018)
	*Shigella dysenterii*	This study, KNIH (Kwak et al., 2018)

1 Multidrug-resistant, indicators, and influenza A/H3N2 virus in this study were provided by the Korea National Institute of Health (KNIH).

Inoculum grown overnight, equivalent to 0.1% of the total volume of the mMRS liquid medium, was utilized for the cultivation of LAB strains. The bacterial inoculum culture was introduced into 4 L of fresh mMRS liquid medium and incubated in a stationary incubator at 30°C for 72 h. The mMRS broth was prepared by combining 2% D-glucose, 0.5% yeast extract, 0.5% sodium acetate (CH_3_COONa), 0.2% ammonium citrate dibasic (C_6_H_14_N_2_O_7_), 0.2% dipotassium hydrogen phosphate (K_2_HPO_4_), 0.005% manganese sulfate monohydrate (MnSO_4_·H_2_O), and 0.01% magnesium sulfate anhydrous (MgSO_4_). It did not contain any peptone. A 3-day culture of *Lb. plantarum* LBP-K10 was utilized to prepare C18-based SPE fractions. To compare the HPLC fractionation patterns, we cultured other isolates, such as *Leuconostoc mesenteroides* LBP-K06 and *Pediococcus pentosaceus* LBP-K17, in the same manner.

Gram-negative and Gram-positive bacterial indicators, as well as multidrug-resistant bacteria, were used to evaluate antibacterial activity. The study utilized multidrug-resistant Gram-positive bacteria *Staphylococcus aureus* 11,471 and *Streptococcus pneumoniae* 14,596, along with the multidrug-resistant Gram-negative bacterium *Salmonella typhimurium* 12,219.

The influenza A (H3N2) virus was employed to induce infection and evaluate the efficacy of antiviral interventions. All bacterial pathogens and the influenza A (H3N2) virus were obtained from the Korea National Institute of Health (KNIH).

### SPE fraction preparation

The culture supernatant of LBP K-10 was concentrated 10-fold prior to freeze-drying. The concentrated supernatant underwent a 24-h liquid-liquid extraction with five times its volume of methylene chloride (CH_2_Cl_2_) employing a separating funnel. Subsequently, the organic phase was lyophilized by evaporation at 55°C. The CH_2_Cl_2_-extracted samples were eventually adjusted to a final volume of 1 mL and underwent SPE using a C18 SPE resin (Waters Sep-Pak C18 Plus cartridge, Millipore Corp., UK). Before this process, the SPE resins were sequentially activated and rinsed with 15 mL of 100% methanol and TDW. Once the sample loading was complete, the SPE resins were equilibrated with TDW. The samples were then loaded onto the SPE resins and eluted using a methanol gradient, increasing in increments of 5% from 5% up to 50%. A stepwise gradient with incremental increases of 5% in methanol concentration was used to facilitate hydrophobic interaction. The eluted solutions were collected and evaporated to remove any remaining organic solvents. The samples were then dissolved in an appropriate amount of TDW for antiviral assays or HPLC fractionation. The resulting samples comprise the following: MeSPEf-5, MeSPEf-10, MeSPEf-15, MeSPEf-20, MeSPEf-25, MeSPEf-30, MeSPEf-35, MeSPEf-40, MeSPEf-45, and MeSPEf-50, representing methanol SPE fractions of 5%, 10%, 15%, 20%, 25%, 30%, 35%, 40%, 45%, and 50%. Unless otherwise specified, this study refers to methanol SPE fractions and methanol SPE eluates as MeSPEfs. Furthermore, the methanol-based SPE experiment is referred to as the MeSPE.

### HPLC

To identify the compounds present in the samples obtained via MeSPE, the MeSPEfs were fractionated using an Agilent prep-HPLC system equipped with CHEMSTATION software. The system utilized a C18 octadecyl bonded silica (ODS) Hypersil column (9.4 mm × 250 mm) with diode array detector (Agilent, USA). The concentration of every fraction obtained from the MeSPEfs was ascertained through prep-HPLC analysis. The column was maintained at a constant temperature of 40°C. The mobile phase consisted of 67% water, 3% acetonitrile (ACN), and 30% methanol. The wavelengths used were 210 nm, 260 nm, and 280 nm. Each fraction obtained from the MeSPEfs was collected, concentrated through evaporation, and then freeze-dried to obtain powder samples.

### Antiviral assay

Antiviral activity was determined using Madin-Darby canine kidney (MDCK) cells as the host cells, as previously described (Kwak et al., 2013). MDCK cells were cultured in complete Dulbecco’s modified Eagle’s medium (DMEM) for the plaque assay. A semi-confluent layer of 500,000 MDCK cells was transferred to six-well culture plates for further cultivation. The cells covered about 90%–95% of the surface area of 90 mm × 15 mm cell culture dishes. After infection with influenza A (H3N2) virus, the cells were cultured for 60 h. Samples in the six-well plates were then inoculated with the virus at a concentration of 2.8 × 10^7^ PFU/mL. To evaluate the plaque-forming assay, we employed *cis*-cyclo(L-Leu-L-Pro) and *cis*-cyclo(L-Phe-L-Pro) analogs, purchased from Bachem (Switzerland), as a reference experiment.

### Antibacterial activity assay

The antibacterial activity of the isolated MeSPEfs was evaluated using established methods. The minimum inhibitory concentration (MIC) was determined using a broth microdilution assay in accordance with the guidelines of the National Committee for Clinical Laboratory Standards (Wikler, 2006). The MeSPEfs were assessed for antibacterial activity against Gram-positive and Gram-negative bacterial indicators, as well as multidrug-resistant bacteria, as previously described (Kwak et al., 2018).

The MeSPEfs were diluted twice to concentrations between 400 and 3.125 μg/mL. After 17-h incubation at 37°C, the MIC was determined. The MeSPEfs’ minimum bactericidal concentration (MBC), defined as the lowest concentration resulting in >99.9% reduction of initial inoculum growth, was measured. The evaluation of antibacterial activity of MeSPEfs utilized bacterial indicators and multidrug-resistant bacteria at a concentration of 5 × 10^5^ CFU/mL (Wiegand et al., 2008).

### Mass analysis

To perform electron ionization (EI) and chemical ionization (CI) of each fraction, we used GC-MS (Agilent, Germany), as described previously (Kwak et al., 2013). A chromatographic system consisting of an Agilent 6,890 series GC equipped with a 7,679 series automatic liquid sampler was used. Mass analysis was performed using a high-resolution mass spectrometer (JEOL JMS-700, Japan).

### Statistical analysis

Data are presented as the mean ± standard deviation (SD) of three independent experiments. One-way analysis of variance (ANOVA) was used for statistical analysis of data gathered during experimental trials to determine the CDP content and antimicrobial activity among the groups. The level of significance was set at *p* = 0.05. All statistical analyses were performed using the STATISTICA 6.0 software package (StatSoft, Tulsa, OK).

## Results and discussion

### The most potent antiviral CF derived from *Lb. plantarum* LBP-K10

**FIGURE 1:**
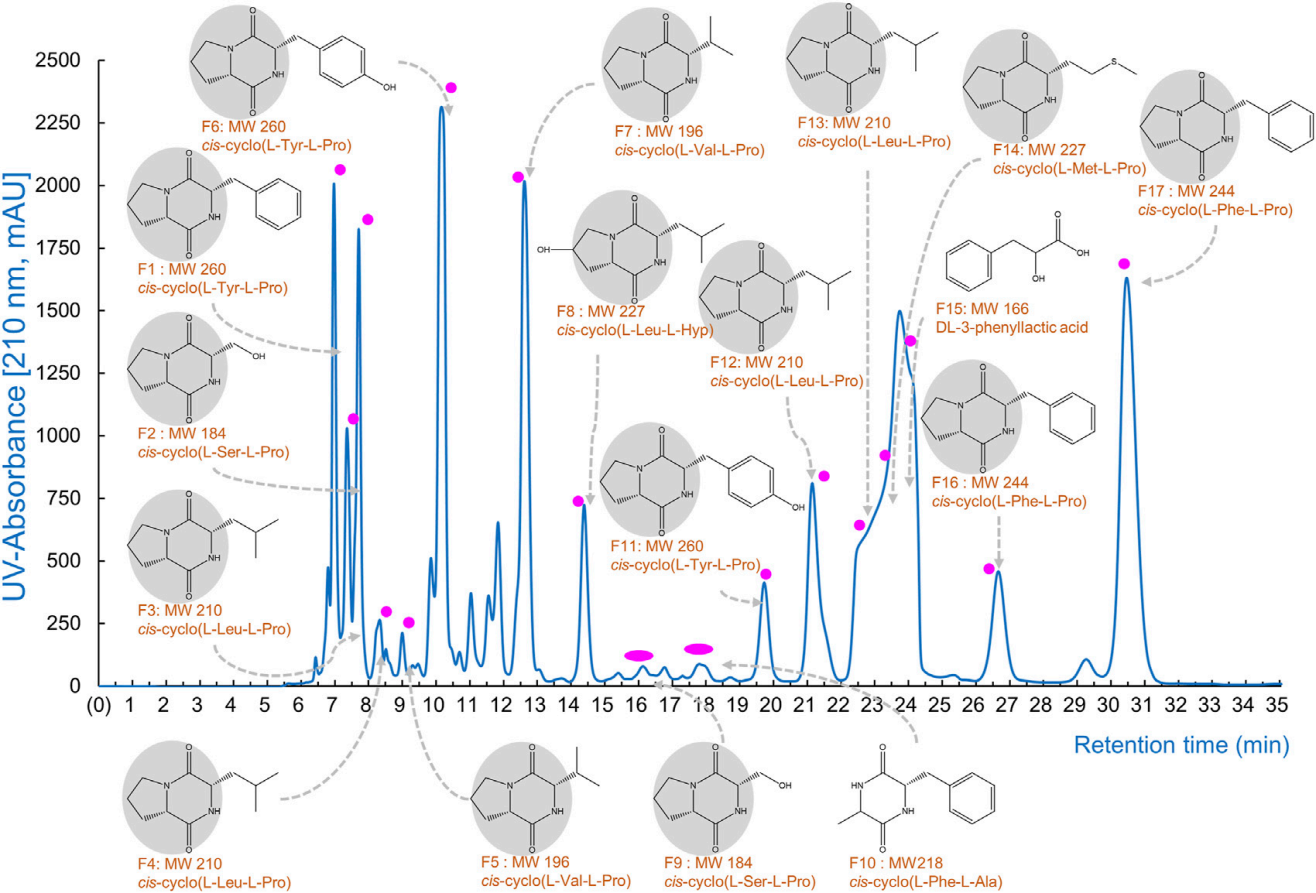
The CDP production by *Lb. plantarum* LBP-K10. The provided figure illustrates the chromatographic profile of the individual CDP fractions acquired from the CF of *Lb. plantarum* LBP-K10. The filtrate obtained following a 3-day growth of LBP K-10 was subjected to extraction using CH_2_Cl_2_ for the purpose of chromatographic separation at a wavelength of 210 nm.

Various CDPs have been achieved using CH_2_Cl_2_-extracted *Lb. plantarum* LBP-K10 CF (Figure 1). Alternatively, combining Amberlite IRA-67 and Purolite A420S AEC resins can also yield CDPs by effectively removing organic acids, sugars, and impurities from *Lb. plantarum* LBP-K10 CF and facilitating the isolation of combined CDP mixtures. These mixtures show noteworthy anti-influenza A (H3N2) viral effects, surpassing those of an individual CDP. Nonetheless, limitations exist in preparing AECs to efficiently obtain CDPs. AECs-derived CDPs demonstrate significantly lower amounts than those acquired from *Lactobacillus* CFs. The AEC process is costly, time-consuming, and generally ineffective in gathering CDPs. From a biosynthetic perspective, naturally occurring CDPs are recognized for their efficacy and biodegradability; nevertheless, their production yield is limited (Tareq et al., 2013; Jia et al., 2019). Laboratory experiments investigating CDPs-microbial induction have encountered certain limitations. While it is possible to produce CDPs from microorganisms using an expression system, the optimization process is time-consuming and not straightforward (Nielsen et al., 2019). To address this issue, we propose a new method to efficiently obtain extremely potent antiviral CDPs via high-throughput C18-based SPE, instead of AECs.

**FIGURE 2:**
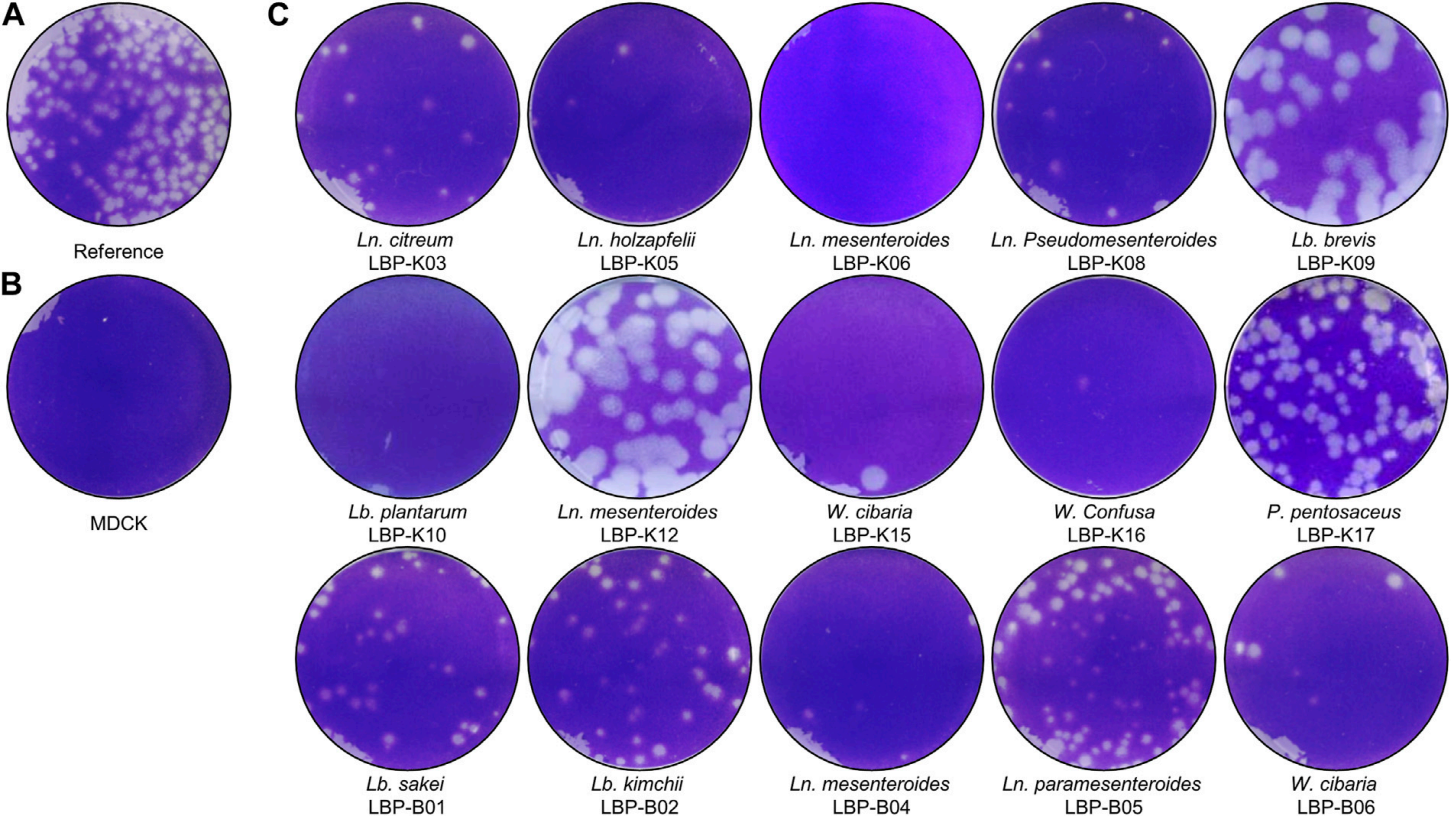
Antiviral activity of isolated LAB. As described in the Materials and Methods section, a solution containing influenza A (H3N2) virus with a concentration of 2.8 × 10^7^ PFU/mL was inoculated into cell cultures labeled as **(A, C)**. In contrast, MDCK cells that were not infected with the virus were designated as **(B)**. In each experimental trial, the samples were diluted by a factor of 0.25 relative to the initial culture, taking into account the total volume of medium utilized in the plaque assay. The LAB samples were treated according to the following procedure: *Ln. citreum* LBP-K03, *Ln. holzapfelii* LBP-K05, *Ln. mesenteroides* LBP-K06, *Ln. Pseudomesenteroides* LBP-K08, *Lb. brevis* LBP-K09, *Lb. plantarum* LBP-K10, *Ln. mesenteroides* LBP-K12, *Weissella cibaria* LBP-K15, *W. Confusa* LBP-K16, *Pediococcus pentosaceus* LBP-K17, *Lb. sakei* LBP-B01, *Lb. kimchii* LBP-B02, *Ln. mesenteroides* LBP-B04, *Ln. paramesenteroides* LBP-B05, and *Weissella cibaria* LBP-B06 are the strains included in the study. All experiments were carried out a minimum of three times.

**FIGURE 3:**
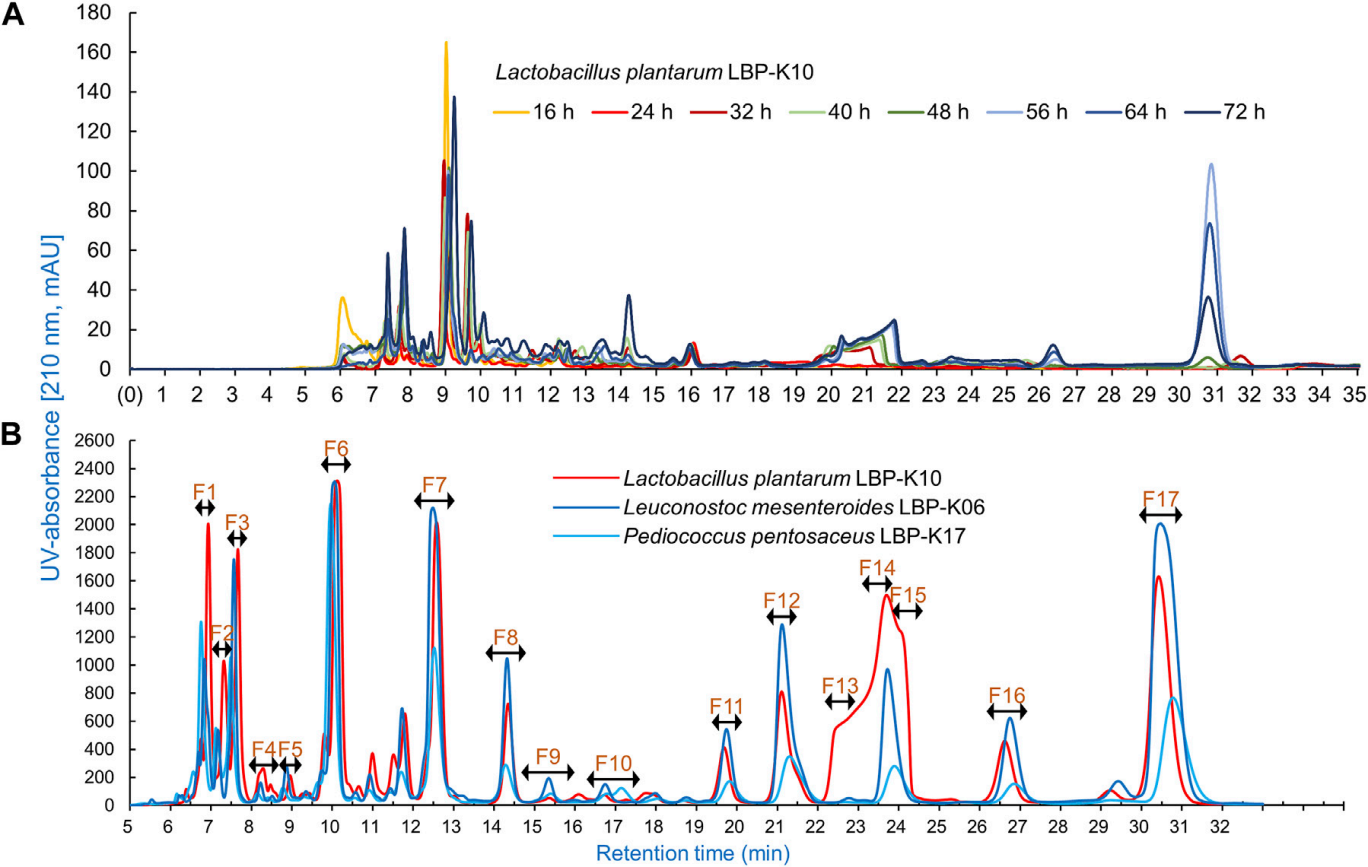
Analytical HPLC chromatogram characteristics of *Lb. plantarum* LBP-K10 CF. **(A)** A significant increase was observed in the production of antimicrobial and antiviral CDPs by *Lb. plantarum* LBP-K10 during different incubation periods. It is worth mentioning that all experiments were performed independently no less than three times. **(B)** The HPLC fractionation pattern of the CFs in *Lb. plantarum* LBP-K10, *Ln. mesenteroides* LBP-K06, and *Pediococcus pentosaceus* LBP-K17 was examined. Chromatographic analysis of the culture filtrates induced by LAB strains was conducted at a wavelength of 210 nm after 72 h. All experiments were performed at least three times independently.

One key experiments in this study observes whether *Lb. plantarum* LBP-K10 CF exhibits higher antiviral activity than other LAB. This comparison is vital before MeSPE as there is no data available on the antiviral activity of all isolated LAB. The plaque-forming assay revealed that solely the CFs from two particular LAB isolates, *Lb. plantarum* LBP-K10 and *Ln. mesenteroides* LBP-K06, displayed anti-influenza virus activity (Figure 2). The HPLC analysis focused on determining the relative peak area of detected fractions and the maximum production of metabolites during *Lb. plantarum* LBP-K10 growth. Before conducting prep-HPLC fractionation, the LAB CFs extracted with CH_2_Cl_2_ were prepared because of their enhanced CDP selectivity during the purification procedure. The peaks of all CFs achieved their peak levels at 72 h when analyzed using analytical HPLC (Figure 3A). In the HPLC chromatogram of *Lb. plantarum* LBP-K10 CF from the 16-h culture, it was observed that the peak area and peak height of the CDPs peak were notably reduced compared to those of *Lb. plantarum* LBP-K10 CF from cultures at different time points. This discrepancy indicates a lack of active CDPs synthesis in the 16-h culture. The peak observed in the preparative high-performance liquid chromatography (prep-HPLC) chromatogram, which aligns with the peak having a retention time of 9 min in the analytical HPLC analysis, corresponds to the peak depicted in Figure 3B, exhibiting a retention time ranging from 9.5 to 10.5 min. This HPLC peak corresponds to the cyclo(Tyr-Pro) of *Lb. plantarum* LBP-K10, which was previously identified in our study through HPLC fractionation and 2D-LC MS/MS analysis, demonstrating antifungal and weak antibacterial properties. Nevertheless, the presence of cyclo(Tyr-Pro) was predominantly noted within the 16-h timeframe, with fluctuations in its quantity observed between 24 and 64 h, albeit without a clearly defined cause. Alternatively, this phenomenon may be attributed to the dual role of microbes as either probiotics or agents of dysbiosis through the secretion of CDPs such as cyclo(L-Leu-L-Pro), cyclo(L-Phe-L-Pro), cyclo(L-Tyr-L-Pro), and cyclo(L-Leu-L-Leu) (Ogilvie and Czekster, 2023). These CDPs have the potential to influence other microbes and their respective hosts. The hydroxyl group of tyrosine, which is derived from the high production of cyclo(Tyr-Pro), is considered more favorable for proton transfer during the initial stages of cell growth compared to the hydroxyl groups of other amino acids like serine and threonine (Schmitt et al., 2018).

In our previous study, the HPLC fractionation patterns generated by CDP production of both *Lactobacillus* genus and other LAB isolates exhibited a high degree of similarity (Kwak et al., 2018; Kang and Kwak, 2024). The production levels of antifungal *cis*-cyclo(L-Val-L-Pro) and antiviral *cis*-cyclo(L-Leu-L-Pro) and *cis*-cyclo(L-Phe-L-Pro) derived from both *Lb. plantarum* LBP-K10 and various previously identified LAB isolates (i.e., *Lb. sakei* LBP-S01, *Lb. plantarum/pentos* LBP-S02, *Lactococcus lactis* LBP-S03, *Lc. lactis* LBP-S06, *Ln. mesenteroides* LBP-K06, *Weissella cibaria* LBP-K15, and *Weissella confusa* LBP-K16) exhibit distinct differences, while their fractionation patterns are consistently similar (Liu et al., 2017; Kwak et al., 2018). This experimental approach, which included the use of HPLC to separate the CH_2_Cl_2_ extracts of bacterial CFs followed by repeated CH_2_Cl_2_ extraction, is distinctive due to its high selectivity for CDP isolation and its adaptability to various modifications of the mobile phases. This procedure typically enables the production of highly pure CDPs suitable for analysis and identification through EI/CI GC-MS as individual compounds, without the presence of any other peptidyl or non-peptidyl molecules. The CDP purification model, in conjunction with CH_2_Cl_2_ extraction, has the capability to generate isolated fractions, all of which exhibit a consistent CDP fractionation pattern in the HPLC chromatograms. The HPLC chromatogram of *Lb. plantarum* LBP-K10 CFs reveals a distinctive broad shoulder in the curved shape of the peak area from F13 to F15 of *Lb. plantarum* LBP-K10, which differs from the sharp peak observed at N13 of *Ln. mesenteroides* LBP-K06 and *P. pentosaceus* LBP-K17 (Kwak et al., 2013; Liu et al., 2017). Moreover, our investigation revealed that the proportion of all fractions derived from the *Lb. plantarum* LBP-K10 CF was greater compared to those from the other isolates. While the CF fractions of *P. pentosaceus* LBP-K17 or *Ln. mesenteroides* LBP-K06 exhibit a lower amount of peak area for CDPs compared to those of *Lb. plantarum* LBP-K10, the identification of CDPs in the CF fractions of *P. pentosaceus* LBP-K17 is notably more straightforward compared to other LAB isolates. In our previous works and studies, *Ln mesenteroides* LBP-K06 and *P. pentosaceus* LBP-K17 have been identified as potentially valuable standardization indicators for CDP research. This indicates that even though there are variations in the substances present in the isolates, LAB could potentially produce and release similar metabolites that act as antiviral agents, such as antifungal and antibacterial CDPs, regardless of the genus of LAB.

Meanwhile, the HPLC peak area of *Lb. plantarum* LBP-K10 CF was significantly higher than that of other isolates (Figure 3B). The chromatographic peak distribution observed in LAB CFs showed remarkable similarities, implying the possible existence of CDPs in the CFs of other isolates, including *Ln. mesenteroides* LBP-K06 and *P. pentosaceus* LBP-K17. Figures 2, 3 illustrate the potential for performing high-throughput MeSPE with CH_2_Cl_2_-extracted *Lb. plantarum* LBP-K10 CF owing to its highly effective antiviral efficacy.

### Significant activity of MeSPEfs against influenza A virus

**FIGURE 4:**
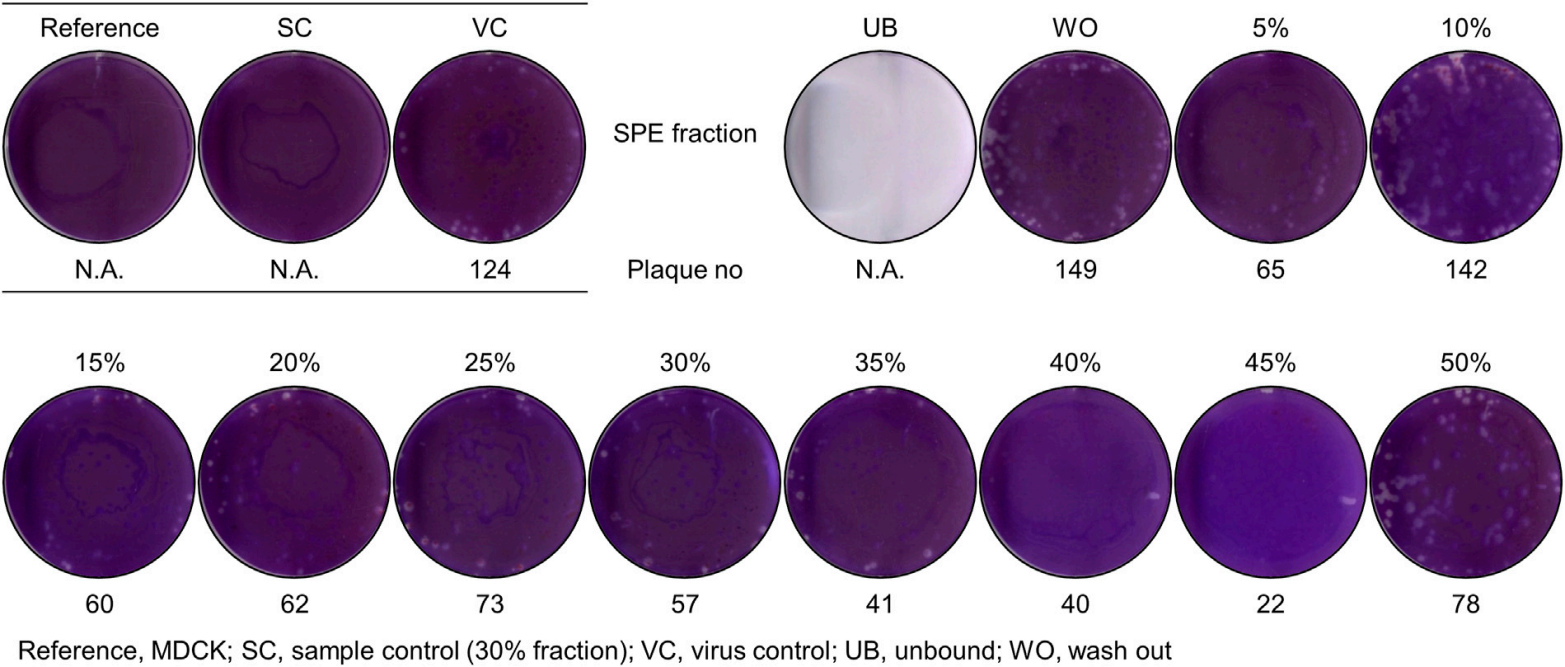
Antiviral activity of MeSPEfs acquired from non-MeSPEfc (CH_2_Cl_2_-extracted *Lb. plantarum* LBP-K10 CF). Before the plaque assay, 2.8 × 107 PFU/mL of influenza A (H3N2) virus was introduced into cell cultures as previously described. A hydrophobic interaction was utilized by employing methanol and a 5% step-gradient to introduce an initial 5% methanol to the SPE resins. The final elution was performed utilizing a 50% methanol solution, following the protocol outlined in the Materials and Methods section. Control experiments were designed and conducted in accordance with established protocols. The abbreviations used in this study are as follows: MDCK, reference; SC, sample control (30% fraction); VC, virus control; UB, unbound; WO, washout. The measured data values are indicative of the mean (± standard deviation) obtained from three separate experiments.

The findings (Figures 2, 3) led to an examination of the antiviral activity of MeSPEfs. To extract substances that were bound to the SPE resin, a 5% increase in methanol concentration was implemented gradually (Figure 4). The CH_2_Cl_2_-extracted *Lb. plantarum* LBP-K10 CF was routinely prepared for MeSPE procedures throughout this study before the plaque-forming assay. Since the CH_2_Cl_2_-extracted *Lb. plantarum* LBP-K10 CF was used for both MeSPE procedures and reference experiments, it has been designated as non-MeSPEfc (CH_2_Cl_2_-extracted *Lb. plantarum* LBP-K10 CF) from this point onwards and throughout the study. MeSPEf-45 showed the most robust effectiveness against the influenza virus, resulting in a noteworthy reduction of up to 82.3% in virus plaque count. While MeSPEf-30, MeSPE-35, and MeSPE-40 demonstrated a decline in plaque count, MeSPEf-45 displayed superior antiviral efficacy, resulting in fewer plaques. MeSPEf-30, MeSPEf-35, and MeSPEf-40 generated 57, 41, and 40 virus plaques, respectively, resulting in 54.0%, 67.0%, and 67.7% reduction in plaque count compared to the control group. The unbound and washout fractions obtained after sample loading were unsuitable for the plaque assay. The low pH effect of each sample is likely responsible for the unbound fraction. The fraction not bound to the SPE resin after undergoing washout with TDW showed a 1.21-fold increase in virus plaques compared to the control experiment. Nevertheless, the cause of this result is unclear since there is no literature or studies to support it. As a result of this finding, further antibacterial activity tests were conducted using MeSPEfs (Table 2). Certain MeSPEfs, specifically MeSPEf-30, MeSPEf-35, MeSPEf-40, and MeSPEf-45, showed exclusive antibacterial activity against bacterial indicators and multidrug-resistant bacteria (Gram-positive *S. aureus* 11,471 and Gram-negative *Salmonella* Typhimurium 12,219). This result shares a noticeable similarity with the antiviral efficacy observed in plaque-forming assays (Figure 4). Figure 4; Table 2 demonstrate the potential effectiveness of high-throughput MeSPE following CH_2_Cl_2_ extraction to target low-molecular-weight compounds. The antiviral activity observed in the plaque assay depicted in Figure 4 aligns with the results obtained from the antagonism test and MIC values presented in Table 2. It is noteworthy that both antibacterial and antiviral activities were detected across the range from MeSPEf-15 to MeSPEf-45, with MeSPEf-45 exhibiting the highest level of activity. The findings suggest variations in the presence and quantity of active substances eluted with methanol among the MeSPEfs samples. MeSPEf-50 exhibited the lowest amount of antimicrobial active substances, while MeSPEf-45 contained significantly higher levels of antibacterial and antiviral active substances.

**TABLE 2:** Relative antibacterial activity of each MeSPEfs isolated from *Lb. plantarum* LBP-K10 CFs.

Strain/methanol %	^3^MeSPEf-5	MeSPEf-10	MeSPEf-15	MeSPEf-20	MeSPEf-25	MeSPEf-30	MeSPEf-35	MeSPEf-40	MeSPEf-45	MeSPEf-50
^1^Antagonism test^a,^*	−	+	+	+	++	++	++	+++	+++	++
^1^Antagonism test^b,^*	−	+	++	++	++	++	++	+++	+++	++
^2^MIC^c,^*	−	−	^4^31.24 ± 1.12	26.55 ± 1.0	20.22 ± 0.58	16.82 ± 3.14	15.92 ± 0.33	11.34 ± 0.63	7.14 ± 0.15	19.81 ± 0.97
^2^MIC^d,^*	−	−	29.05 ± 0.95	25.11 ± 1.75	19.04 ± 0.64	15.06 ± 1.27	13.21 ± 1.13	10.58 ± 0.78	9.525 ± 0.88	20.48 ± 1.05
^1^Antagonism test^e,^*	−	+	+	+	++	++	++	+++	+++	++
^1^Antagonism test^f,^*	−	+	++	++	++	++	++	+++	+++	++
^2^MIC^g,^*	−	−	31.24 ± 1.12	26.55 ± 1.0	20.22 ± 0.58	16.82 ± 3.14	15.92 ± 0.33	11.34 ± 0.63	7.14 ± 0.15	19.81 ± 0.97
^2^MIC^h,^*	−	−	29.05 ± 0.95	25.11 ± 1.75	19.04 ± 0.64	15.06 ± 1.27	13.21 ± 1.13	10.58 ± 0.78	9.525 ± 0.88	20.48 ± 1.05

1 Symbol: +, <15 mm; ++, <22 mm; +++, >22 mm (Indicator strains: *Bacillus subtilis* ^*a*^.

*E. coli* ^*b*^; multidrug-resistant bacteria: *S. aureus* 11,471 ^*e*^.

*S*. Typhimurium 12,219 ^*f*^).

2 MIC: Minimum inhibitory concentration (Indicator strains: *B. subtilis* ^*c*^.

*E. coli* ^*d*^; multidrug-resistant bacteria: *S. aureus* 11,471 ^*g*^.

*S*. Typhimurium 12,219 ^*h*^).

3 Samples include: MeSPEf-5, MeSPEf-10, MeSPEf-15, MeSPEf-20, MeSPEf-25, MeSPEf-30, MeSPEf-35, MeSPEf-40, MeSPEf-45, and MeSPEf-50, which represent methanol SPE, fractions of 5%, 10%, 15%, 20%, 25%, 30%, 35%, 40%, 45%, and 50%.

4 Data are presented as mean ± standard error of the mean from three independent experiments.

Several simple CDPs have been reported to possess antibacterial activity against both Gram-negative and Gram-positive bacteria, although with relatively low potency levels (Milne et al., 1998; Rhee, 2002). In addition, cyclo(L-Leucine-L-Proline) has shown activity against 12 vancomycin-resistant enterococci (VRE) bacterial strains, while cyclo(L-Phenylalanine-L-Proline) has demonstrated similar activity against VRE strains and other pathogenic microbes (Rhee et al., 2001; Rhee, 2002). Nevertheless, the combination of X and Y appears to have a synergistic effect. The drug combination is highly effective against anaerobic Gram-negative and Gram-positive bacteria, surpassing the efficacy of other antimicrobial agents such as erythromycin, cefoxitin, imipenem, clindamycin, and metronidazole significantly. Additionally, it demonstrated an MIC_50_ value of 0.064 mg/L against *Clostridium difficile* (Rhee, 2006). Moreover, bicyclomycin derivatives possessing CDP structures sourced from *Streptomyces sapporonesis* and *S. aizumenses* (Borthwick, 2012), along with proline-based CDPs functioning as both quorum sensing agonists and antagonists, are demonstrating promising clinical effectiveness (Degrassi et al., 2002; Park et al., 2006). Proline-based compounds show potential for treating infections caused by major human pathogens (Li et al., 2011; Gowrishankar et al., 2016). Their non-bactericidal mode of action offers an appealing alternative to traditional antibiotics (Gowrishankar and Karutha Pandian, 2017). However, understanding the intricate microbial networks and metabolites related to homeostasis and dysbiosis is crucial for predicting their effects on microbes associated with health or disease.

Consequently, this study focuses on isolating small cyclic dipeptidyl compounds through high-throughput MeSPE. Nonetheless, this approach does not encompass proteinaceous fractions such as bacteriocins. This experimental design has practical implications for class IIa bacteriocins, which are potentially useful as antiviral agents but have certain limitations (AI Kassaa et al., 2014; Alvarez-Sieiro et al., 2016; Dey et al., 2021). These constraints arise from necessary and specific conditions, including temperature, pH, nutrient availability, and salt concentration (De Vuyst and Leroy, 2007; Lohans and Vederas, 2012). To increase the potency of class IIa bacteriocins, it is crucial to eliminate any N-terminal disulfide bridges. Nevertheless, substituting these bonds with carbocycles produces biologically active peptides, but their activity decreases by tenfold. These findings contradict the ease of isolating antiviral peptidyl metabolites like tripeptides (Hu et al., 2021), dipeptides (Ceña-Diez et al., 2023), and 2-5-diketopiperazines (Huang et al., 2014).

### The discrepancies observed in fractions obtained via HPLC fractionation between MeSPEfs and non-MeSPEfc (CH_2_Cl_2_-extracted *Lb. plantarum* LBP-K10 CF)

**FIGURE 5:**
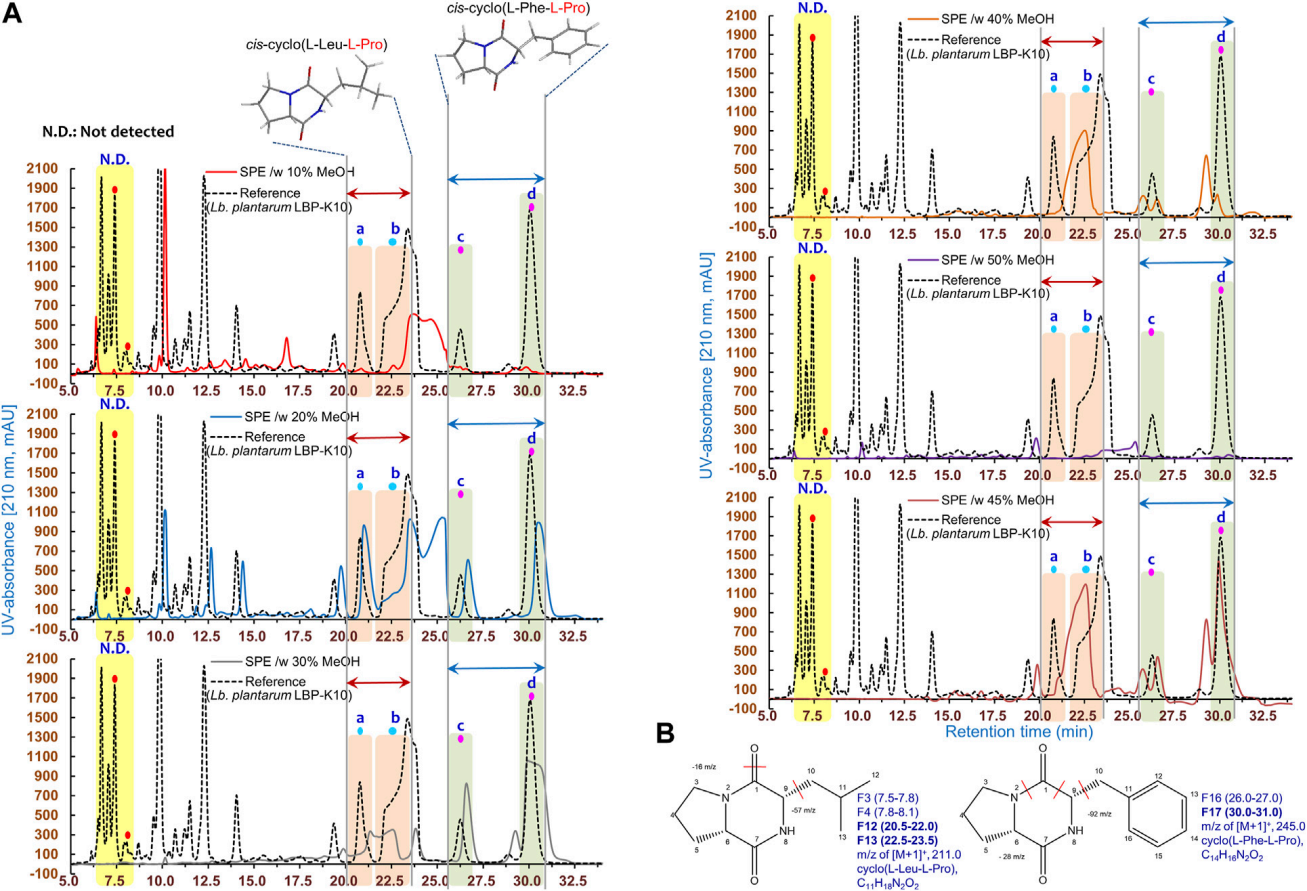
HPLC chromatograms of the samples obtained through SPE from *Lb. plantarum* LBP-K10 CF. **(A)** The MeSPEfs in the HPLC experiment were classified based on the methanol concentration used for elution. The HPLC chromatogram of non-MeSPEfc (CH_2_Cl_2_-extracted *Lb. plantarum* LBP-K10 CF) presents a comparison of CDP fractions. In every HPLC chromatogram, the terms a and b represent the fractions of *cis*-cyclo(L-Leu-L-Pro) with a retention time between 20.5 and 23.5 min, derived from non-MeSPEfc (CH_2_Cl_2_-extracted *Lb. plantarum* LBP-K10 CF) fractions F12 and F13. The terms c and d refer to the fractions *cis*-cyclo(L-Leu-L-Pro) with a retention time of 26.1–31.3 min, derived from fractions F16 and F17 of non-MeSPEfc (CH_2_Cl_2_-extracted *Lb. plantarum* LBP-K10 CF). **(B)** Information regarding these proline-containing CDPs originating from *Lb. plantarum* LBP-K10 is extensively discussed in the main body of the text. Each experiment is depicted by individual lines, which correspond to the observed peaks. Each experiment was conducted at least three times, independently.

Based on the findings of the plaque-forming assay (Figure 4), we examined the HPLC fractionation patterns of MeSPEfs and non-MeSPEfc (CH_2_Cl_2_-extracted *Lb. plantarum* LBP-K10 CF). In a previous section, it was mentioned that the utilization of initial CH_2_Cl_2_ extraction was carried out to obtain *Lb. plantarum* LBP-K10 CF for both MeSPEfs and non-MeSPEfc (CH_2_Cl_2_-extracted *Lb. plantarum* LBP-K10 CF). This process ensured experimental uniformity and enabled easier detection of cyclic dipeptidyl molecules. The HPLC chromatogram displayed the patterns of fractionation for MeSPEfs, which were eluted in increments that varied by 10% in methanol concentration (Figure 5). The majority of the fractions separated from MeSPEfs had minimal peak detection in the first half of the chromatogram, particularly in the retention time (*t*_*R*_) range spanning from 5 to 20 min. The detectable peaks of the MeSPEfs were predominantly evident in the later segment of the *t*_*R*_ range (16.0-33.5). The peak positions showed a certain level of dependence on the methanol concentration, with the exception of MeSPEf-50. However, none of the fractions separated from MeSPEf-10 and MeSPEf-20 were in line with the fractionation pattern observed in the non-MeSPEfc (CH_2_Cl_2_-extracted *Lb. plantarum* LBP-K10 CF). A critical factor in characterizing antiviral fractions acquired via MeSPEfs was the peak-to-peak correspondence between MeSPEfs and non-MeSPEfc (CH_2_Cl_2_-extracted *Lb. plantarum* LBP-K10 CF) (Figure 5). A peak-to-peak alignment strategy was used to enhance the efficiency of data analysis. Analysis of the peak-to-peak alignment between MeSPEfs and non-MeSPEfs revealed no significant correlation. The peak-to-peak correspondence observed between MeSPEfs and non-MeSPEfc (CH_2_Cl_2_-extracted *Lb. plantarum* LBP-K10 CF), exclusively in the middle to latter portions of the *t*_*R*_ range, suggests the presence of specific antiviral effectors that selectively attack the influenza virus. Comparable yet noteworthy results were noted for the peaks exclusively detected in the MeSPEfs associated with *cis*-cyclo(L-Leu-L-Pro) and *cis*-cyclo(L-Phe-L-Pro), which corresponded with non-MeSPEfc (CH_2_Cl_2_-extracted *Lb. plantarum* LBP-K10 CF) fractions F13 and F17. Nevertheless, the initial HPLC separation of MeSPEfs revealed peaks that were asymmetric and overlapping, exhibiting absorption at 210 nm (Figure 5). The retention times of these peaks did not correspond to those of any identified CDP fraction from the previously examined fractions F13 and F17 of *Lactobacillus* CDPs’ fractions F13 and F17, *cis*-cyclo(L-Leu-L-Pro) and *cis*-cyclo(L-Phe-L-Pro), respectively. The aforementioned fractions F13 and F17 demonstrated the highest level of antimicrobial efficacy in our previous work (Kwak et al., 2018). Most fractions of MeSPEfs showed lower peak areas associated with *cis*-cyclo(L-Leu-L-Pro) (corresponding to non-MeSPEfc (CH_2_Cl_2_-extracted *Lb. plantarum* LBP-K10 CF) F3, F4, and F12 fractions) and *cis*-cyclo(L-Phe-L-Pro) (corresponding to non-MeSPEfc F16 fraction) in comparison to the non-MeSPEfc control. To provide definitive support for potent and targeted antiviral combinations, we conducted a comparative analysis of MeSPEfs plaque-forming assay results (Figure 4) and their HPLC fractionation pattern (Figure 5). This assumption was based on the peak-to-peak correspondence between MeSPEfs and non-MeSPEf chromatograms within the *t*_*R*_ range’s middle to latter parts. Certain chromatograms of MeSPEfs, specifically MeSPEf-30, MeSPEf-35, MeSPEf-40, and MeSPEf-45, exhibited significant skewness towards the latter portion of the chromatogram in terms of *t*_*R*_. Consequently, the results of the plaque-forming assay suggest that there may be specific MeSPEfs present with the aforementioned effectors.

**FIGURE 6:**
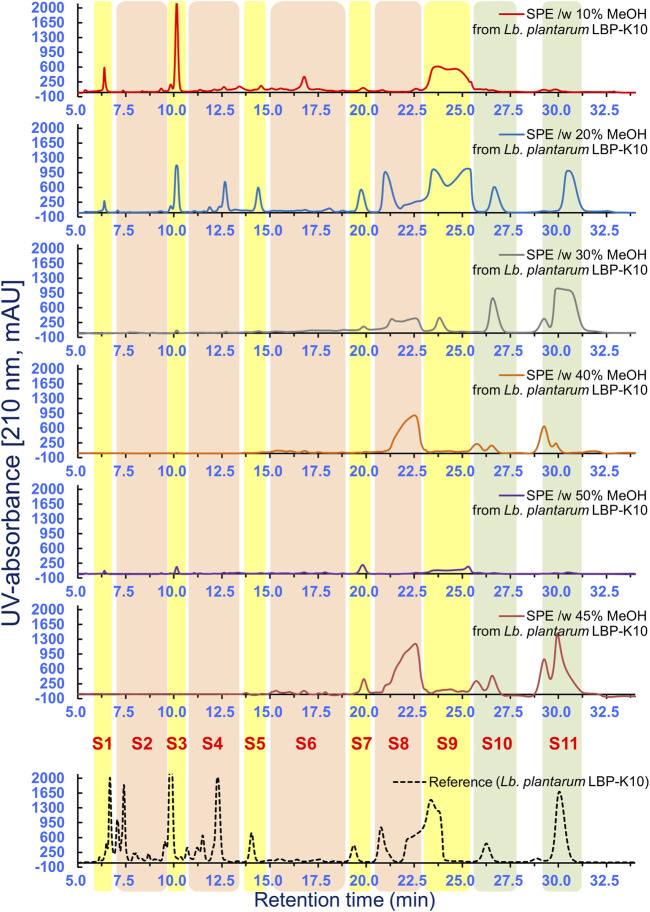
HPLC separation of all MeSPEfs. A further HPLC fractionation method was developed by utilizing samples obtained through MeSPE with methanol elution at 5% intervals, ranging from 5% to 50%. Fractionation was subsequently performed to gather 11 fractions from each MeSPEf. The results obtained in this experiment were derived from a minimum of three independent experiments.

Several peaks in MeSPEfs fused together, depending on the degree of overlap. For instance, the fractionation pattern of the overlapping HPLC peaks in the MeSPEfs samples posed challenges in identifying the specific HPLC peaks corresponding to F13 and F17 of *Lb. plantarum* LBP-K10. To address this issue, overlapping peaks that might impact each other were effectively separated by employing distinct isocratic mobile-phase compositions at various pH levels. This approach allowed for the differentiation of primary peaks before precise estimations of any parameter could be made. Due to endeavors aimed at isolating individual peaks from intricate chromatograms (Figures 5, 6), we successfully isolated a singular compound with the highest antiviral activity from all types of peaks obtained from MeSPEf-45. This was achieved by meticulously controlling the experimental parameters related to elution volume at peak maximum and peak height.

### Antibacterial and antiviral activities of fractions fractionated from MeSPEfs

As described, in addition to the lack of a noticeable peak-to-peak correlation between MeSPEfs and non-MeSPEfc (CH_2_Cl_2_-extracted *Lb. plantarum* LBP-K10 CF) (Figure 5), a major portion of MeSPEfs fractions displayed skewness towards the latter part of the chromatogram. This discovery holds significance since fractions from later sections of the chromatograms may contain antiviral substances in substantially higher quantities than fractions from the former section. As previously stated, the fractionation pattern of the majority of the MeSPEfs during the resolution of co-eluting peaks into individual components suggested the potential presence of CDPs in nearly all fractions of MeSPEfs, given the selective observation of HPLC peaks corresponding to CDPs in non-MeSPEfc (CH_2_Cl_2_-extracted *Lb. plantarum* LBP-K10 CF). Another supporting factor for this assumption was observed in the latter part of the HPLC profile of non-MeSPEfc (CH_2_Cl_2_-extracted *Lb. plantarum* LBP-K10 CF). In this section, the HPLC peaks corresponding to F13 [cis-cyclo(L-Leu-L-Pro)] and F17 [cis-cyclo(L-Phe-L-Pro)], which are the antiviral CDPs of this particular strain, were identified in the fractionation pattern of the MeSPEfs samples. This identification was achieved by consolidating their overlapping peaks into a singular peak during the separation process.

Hene, HPLC fractionation was carried out to separate and collect individual fractions separated from MeSPEfs (Figure 6) in light of the antibacterial and antiviral activity findings. The aim was to re-evaluate the antiviral activity to pinpoint potential targets for antiviral substances. Through antibacterial assays (Supplementary Table S1) and antiviral experiments (Table 3), we elucidated the distinct impacts of specific fractions fractionated from MeSPEfs on pathogenic microbes. The results suggest that fractions obtained from MeSPEf-45 display superior antiviral efficiency against the influenza virus in contrast to fractions isolated from other MeSPEfs. This increased activity is likely caused by the higher amounts of S8 and S11 fractions. The results were also notable for their significant antibacterial effects. The activity against the Influenza A (H3N2) virus (Table 3) and antibacterial activity (Supplementary Table S1) were solely observed in two of the eleven collected fractions, S8 and S11. No antiviral activity or antibacterial antagonism was observed in any of the assessed fractions, except for fractions S8 and S11.

**TABLE 3:** The fractions separated from MeSPEfs were evaluated for their anti-influenza A virus (H3N2) activity.

Control	Plaque number (±SD) of non-MeSPEfc (CH_2_Cl_2_-extracted *Lb. plantarum* LBP-K10 CF)										
^*1*^ Ref	^*4*^ 0										
^*1*^ VC	122.1 ± 11.3										
^*1*^ UB	0										
^*1*^ WO	140.0 ± 9.50										
Fraction no.											
SPE Sample	^*5*^ S1	S2	S3	S4	S5	S6	S7	S8	S9	S10	S11
	^*6*^ 6.0–7.0	7.1–9.4	9.5–10.5	10.6–13.4	13.5–15.0	15.1–17.5	17.6–20.4	20.5–23.0	23.1–26.0	26.1–28.0	29.0–31.3
	Plaque Number (±SD) of MeSPEfs										
^*2,3*^ MeSPEf-5	^*7*^ N.S.	N.S.	N.S.	N.S.	N.S.	N.S.	N.S.	N.S.	N.S.	N.S.	N.S.
MeSPEf-10	N.S.	N.S.	N.S.	N.S.	N.S.	N.S.	N.S.	N.S.	N.S.	N.S.	N.S.
MeSPEf-15	N.S.	N.S.	N.S.	N.S.	N.S.	N.S.	N.S.	101.3 ± 10.2	N.S.	N.S.	N.S.
MeSPEf-20	N.S.	N.S.	N.S.	N.S.	N.S.	N.S.	N.S.	81.5 ± 6.50	N.S.	N.S.	89.30 ± 14.0
MeSPEf-25	N.S.	N.S.	N.S.	N.S.	N.S.	N.S.	N.S.	86.0 ± 4.13	N.S.	N.S.	91.5 ± 9.63
MeSPEf-30	N.S.	N.S.	N.S.	N.S.	N.S.	N.S.	N.S.	N.S.	N.S.	96.5 ± 10.45	75.0 ± 8.50
MeSPEf-35	N.S.	N.S.	N.S.	N.S.	N.S.	N.S.	N.S.	98.0 ± 7.25	N.S.	N.S.	66.3 ± 5.73
MeSPEf-40	N.S.	N.S.	N.S.	N.S.	N.S.	N.S.	N.S.	68.0 ± 6.55	N.S.	N.S.	N.S.
MeSPEf-45	N.S.	N.S.	N.S.	N.S.	N.S.	N.S.	N.S.	54.3 ± 9.14	N.S.	105.3 ± 11.1	51.0 ± 4.80
MeSPEf-50	N.S.	N.S.	N.S.	N.S.	N.S.	N.S.	N.S.	N.S.	N.S.	N.S.	N.S.

^a^ Reference, MDCK; VC, virus control; UB, unbound; WO, wash out.

^b^ MeSPEfs, eluted with different concentration of methanol.

^c^ Samples include: MeSPEf-5, MeSPEf-10, MeSPEf-15, MeSPEf-20, MeSPEf-25, MeSPEf-30, MeSPEf-35, MeSPEf-40, MeSPEf-45, and MeSPEf-50, which represent methanol SPE, fractions of 5%, 10%, 15%, 20%, 25%, 30%, 35%, 40%, 45%, and 50%.

^d^ Data are presented as mean ± standard error of the mean from three independent experiments.

5 Fractions of MeSPEfs, eluted with different methanol concentrations.

6 Retention Times (min).

The term “^*7*^ N.S.” in this context, the term “non-significant” is relevant to two distinct scenarios. The initial situation occurs when the number of plaques obtained in the plaque assay surpasses the quantity of plaques in the virus control (VC). The second scenario arises when there is a decrease in the quantity of plaques, but the variance is below 10%.

Based on the antiviral activity of the two fractions of MeSPEf-45, we purified 11 individual fractions from MeSPEf-45 for antiviral activity assays. It is worth noting that in this study, the majority of MeSPEf-45 fractions were not separated as a single compound. For instance, the HPLC separation of fraction S2 revealed peaks that were both asymmetric and overlapping, with absorption observed at 210 nm. Several peaks in fraction S2 merged together and could have potentially affected each other due to the high degree of overlap. The precise assessment of any given parameter necessitated differentiation of the primary peaks through different isocratic mobile-phase compositions at varying pH levels. Unfortunately, this adjustment failed to effectively differentiate the overlapping peaks, leading to imprecise analysis. Despite our extensive efforts to isolate individual peaks from the complex chromatograms, we were unable to isolate any compounds from fraction S2. We meticulously controlled the experimental parameters of elution volume at peak maximum and peak height, yet no other fractions showed any compounds except for S8 and S11.

### Characterization of antiviral fractions containing bioactive CDPs from MeSPEfs

The use of CI mode proves advantageous when the molecular ion peak is missing in EI mode, particularly for identifying samples of unknown origin. Nevertheless, in this study using EI/CI GC-MS, 9 fractions from MeSPEf-45 remained unidentified. Besides the purified fractions S8 and S11, we did not observe any molecular ions in their low-quality EI mass spectra. Fragment ions were not clearly observed due to uncertain molecular ions and the lack of high-resolution mass measurements. EI and CI fragmentation yielded poor results with confirmatory ions <10% of the base peak. Under these circumstance, the EI and CI fragmentation did not reveal significant information to distinguish between them. The sensitivity of EI/CI GC-MS was insufficient to accurately determine the molecular mass. This inadequacy resulted from a combination of poor ionization patterns and limited signals across most MeSPEfs fractions (data not shown). The primary culprit was the overlapping of peaks owing to insufficient chromatographic fractionation. This result indicates that the overlapped peaks may comprise various types of compounds that cannot be differentiated.

**FIGURE 7:**
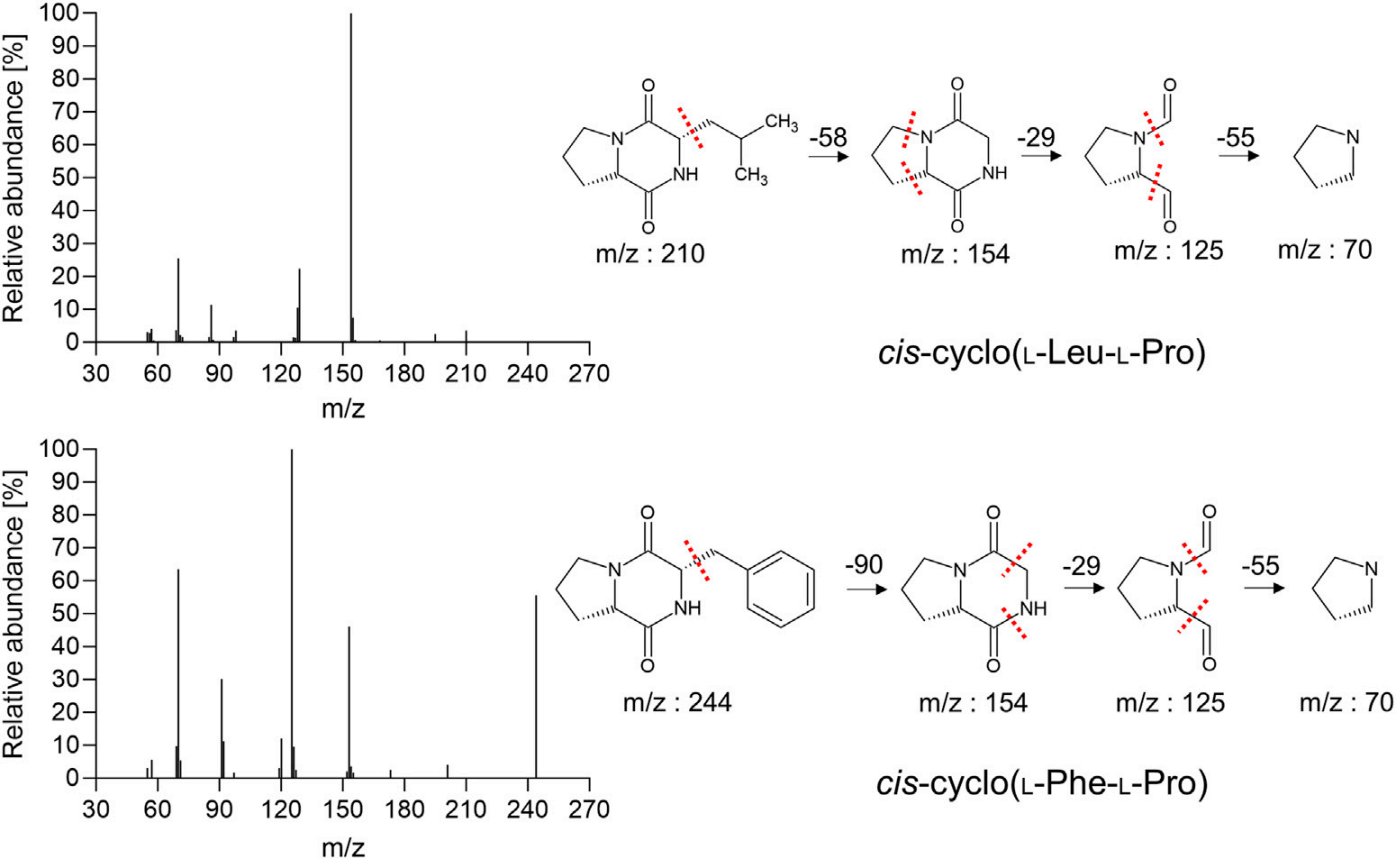
The CDPs that were identified in fractions S8 and S11 from MeSPEf-45. EI fragmentation patterns are displayed with dashed lines marking the separation of structural units by the chemical bonds. The patterns are shown twice for clarity.

The lyophilized powders of the isolated fractions S8 and S11 underwent structural examination using EI/CI GC-MS. The molecular ion peaks at *m/z* 211 and *m/z* 244, indicating both [M+1]^+^, respectively (Figure 7; Table 4). EI and CI fragmentation patterns revealed that fractions S8 and S11 of MeSPE-45, which showed a substantial increase compared to other MeSPEfs in the chromatogram, represent *cis*-cyclo(L-Leu-L-Pro) and *cis*-cyclo(L-Phe-L-Pro), identified as C_11_H_18_N_2_O_2_ and C_14_H_16_N_2_O_2_, correspondingly. The identified mass spectra of the chromatographic peaks offer proof of the *cis*-cyclo(L-Leu-L-Pro) and *cis*-cyclo(L-Phe-L-Pro), which display antiviral properties. Thus, it seems likely that MeSPEfs, consisting of two antiviral proline-based CDPs, can effectively treat viral infections.

**TABLE 4:** Mass spectrometric analysis was conducted on fractions S8 and S8 obtained from MeSPEf-45 using EI GC-MS.

Fractions	m/z of [M+1]^+^	m/z (%) of EI-MS	Predicted molecules
S8 (20.5–23.0)^a^	211.0	210.0 (3.5), 195.0 (2.5), 168.0 (0.5), 156.0 (0.7), 155.0 (7.5), 154.0 (100.0), 129.0 (22.4), 128.0 (10.5), 127.0 (1.4), 126.0 (1.5), 98.0 (3.5), 97.0 (1.6), 87.0 (0.8), 86.1 (11.4), 85.0 (1.6), 72.0 (1.6), 71.0 (2.2), 70.0 (25.5), 69.0 (3.6), 58.0 (0.5), 57.0 (4.1), 56.0 (2.8), 55.0 (3.1), 54.0 (1.5)	*cis*-cyclo(L-Leu-L-Pro), C_11_H_18_N_2_O_2_
S11 (29.0–31.3)^a^	245.0	244 (55.5), 201 (4.1), 173.2 (2.5), 155.1 (1.7), 154.1 (3.6), 153.1 (46.1), 152.1 (2.0), 127.2 (2.5), 126.2 (9.5), 125.2 (100), 120.2 (12.0), 119.1 (3.0), 97.1 (1.7), 92.1 (11.2), 91.1 (30.1), 71.1 (5.4), 70.1 (63.5), 69.1 (9.7), 57.1 (5.5), 55.0 (3.0)	*cis*-cyclo(L-Phe-L-Pro), C_14_H_16_N_2_O_2_

^a^ Retention time (min).

Importantly, no conformational enantiomeric pairs or diastereomeric structures were found in any of the fractions acquired from the MeSPE method in the current study. It is noteworthy that these findings align with prior research on proline-based CDPs, but do not pertain to the current study. Combining enantiomers or diastereomers leads to synergistic antimicrobial effects, which are more potent than those of a single compound. These structural variances occur due to intramolecular hydrogen bonding within the cyclic dipeptidyl moiety. The investigations of enantiomeric pairs and diastereomeric structures highlight a possible connection between the molecular structures of established compounds, including CDPs, and their *t*_*R*_ range (Shan et al., 2008; Ishikawa et al., 2010). The proline-based CDPs frequently occur as conformational enantiomeric pairs or diastereomeric structures, leading to alterations in *t*_*R*_ and the emergence of unique peaks in the HPLC chromatogram (Swadesh, 2000; Lesma et al., 2012). *Cis*-cyclo(L-Leu-L-Pro) appears in multiple regions of the chromatogram, particularly in the non-MeSPEfc (CH_2_Cl_2_-extracted *Lb. plantarum* LBP-K10 CF) fraction F13, corresponding to the latter portion of S8. It is also detected in non-MeSPEfc fraction F3 (*t*_*R*_, 7.5-7.8) and non-MeSPEfc fraction F4 (*t*_*R*_, 7.8-8.1), likely corresponding to S2. Furthermore, the non-MeSPEfc fraction F12 (*t*_*R*_, 20.5-22.0) is related to the former part of S8. Since S11 contains *cis*-cyclo(L-Phe-L-Pro) according to the EI/CI GC-Ms data, the S10 (*t*_*R*_, 26.1-28.0) that corresponds to the non-MeSPEfc (CH_2_Cl_2_-extracted *Lb. plantarum* LBP-K10 CF) fraction F16 is likely to suggest the existence of *cis*-cyclo(L-Phe-L-Pro). Nevertheless, we failed to establish a correlation between MeSPEfs fractions and enantiomeric pairs or diastereomeric structures because of the overlapping peaks’ extreme difficulty in distinguishing, leading to imprecise analysis, as previously explained.

The study findings indicate that numerous trials are necessary to optimize the methodological effectiveness of the MeSPE technique. The extraction of peptides from the biological matrix is a crucial initial procedure before chromatographic separation and mass spectrometry detection or radio-metabolite profiling (Ewles and Goodwin, 2011; Katsila et al., 2012). For instance, the optimal extraction conditions for highly hydrophilic peptides, such as somatostatin catabolites, may differ significantly from those suitable for hydrophobic peptides, as exemplified by liraglutide. When employing SPE and simple extraction methods utilizing methanol and ethanol, as well as 80% methanol and 80% ethanol solutions, researchers have utilized these solvents to extract compounds for the assessment of antioxidant activity and phenolic compounds in oils. The results indicate that the methanolic extracts of hemp and milk thistle seed oils exhibit the highest antiradical activity, while the ethanolic extracts demonstrate superior reducing properties (Kalinowska et al., 2022). Polar peptides are typically more compatible with solvents such as methanol and ethanol, while hydrophobic peptides may require aprotic solvents like acetonitrile. The proportion of matrix to solvent volume (usually varying between 1:2 to 1:5) and the incorporation of acids (e.g., formic or trifluoroacetic acid) or bases (e.g., ammonia) in the protein precipitation process can markedly impact the efficiency of the recovery process (Esposito et al., 2020). The ability of an elution solvent to dissolve particular target analytes should be directly proportional to their subsequent retrieval. The elution solvent for SPE experiments was identified by assessing the recovery rates with different solvent systems, such as methanol, acetonitrile, methanol:acetonitrile (1:1), dichloromethane, and acetonitrile:dichloromethane (1:1). Various volumes between 2 and 5 mL can be examined to assess solvent recoveries, and the most suitable volume should be chosen for further studies (Liang et al., 2016). In the context of developing purification methods for radiopharmaceuticals intended for clinical use, the timely integration of biocompatible solvents provides advantages and aids in addressing potential challenges that may arise during subsequent stages of the process. For example, the utilization of ethanol in the development of HPLC methods as opposed to methanol. The use of ethanol in HPLC has been associated with elevated back pressure, requiring modifications in flow rate and column parameters (such as stationary phase and dimensions) to maintain the highest level of purification effectiveness. Furthermore, replacing trifluoroacetic acid with an alternative acid, such as hydrochloric, acetic, or phosphoric acid, may be considered suitable for pH adjustment (Barnes et al., 2022).

### The combined fractions S8 and S11 with increased activity against pathogenic microbes and viruses compared to a single fraction separated from MeSPE-45

As demonstrated by both Figure 4, which depicts antiviral activity when using MeSPEf, and the MIC experiments in Table 2, the MeSPEf itself has inherent antibacterial activity. When utilized as the experimental group, the MeSPEf, which represents a mixture of all peaks in the sample, exhibits a wide range of antibacterial and antiviral activities across different samples, ranging from 15% to 50%. However, variations exist among the MeSPEfs. The fractionated provide insight into the specific fractions that contribute to the antibacterial and antiviral activity, compared to the experimental group that utilized the MeSPEfs directly. Figures 4–6, along with Tables 2 and Supplementary Table S1, depict the core characteristics of the MeSPE-obtained eluate. According to our findings, fractionation of MeSPEfs permits the comprehensive identification of individual fractions at different methanol concentrations. This provide insight into the precise antiviral and antibacterial properties of each fraction. We posit that a synergistic bioactive formula can be developed by combining fractions from MeSPEf-45, containing compounds with both antibacterial and antiviral activities.

**FIGURE 8:**
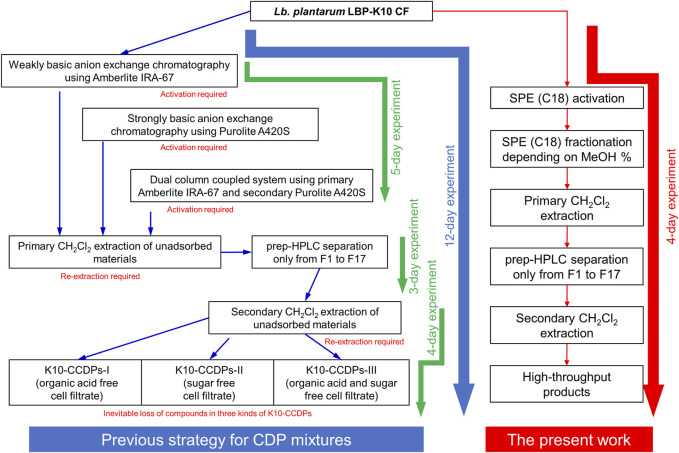
Comparisons between the SPE technique and previously demonstrated AECs. Significantly improved efficiency in terms of both yield and time is attained when employing SPE resin for the isolation of CDPs from LAB cultures, in comparison to AEC resins such as Amberlite IRA-67, Purolite A420S, or a combination of both. The presented data demonstrates a more efficient approach to purifying a substantial quantity of combined CDPs. The application of MeSPE techniques in modified antiviral CDP combinations has led to significant experimental progress, providing valuable insights into potential enhancements for the efficacy of antiviral treatments.

We established the efficacy of the high-throughput MeSPE approach by conducting a thorough evaluation of the antimicrobial properties using fractions obtained from MeSPEf-45 in specific combinations. We assessed the antiviral activity of the combined fractions using a plaque-forming assay (Table 5), following the evaluation of the antibacterial effectiveness of MeSPEf-45 (Supplementary Table S2). As anticipated, most experimental groups showed an increase in the anti-influenza A virus effect that relied on the fraction combination when using MeSPEf-45. Similarly, a proportional boost in the antibacterial activity against Gram-positive, Gram-negative, and multidrug-resistant bacteria was noted when using the combined fractions acquired from MeSPEf-45. The MeSPE techniques are essential for achieving optimal performance as they eliminate any need for additional and time-consuming processing procedures. Hence, the anti-influenza A virus properties of the MeSPEf or any subsequently fractionated fraction can be harnessed through selective employment of S8 and S11. The combination of SPE resin with *Lb. plantarum* LBP-K10 CF shows potential to enable efficient and selective biosynthesis of *cis*-cyclo(L-Leu-L-Pro) and *cis*-cyclo(L-Phe-L-Pro). However, MeSPEf-45, non-fractionated MeSPEf, shows higher levels of antiviral activity against Influenza A virus than other MeSPEfs and their fractionated fractions. For this reason, it is recommended to avoid further secondary fractionation in order to enhance the efficiency of obtaining antiviral fractions. As previously mentioned, despite various efforts, all of the preceding fractions of the HPLC chromatogram, including the S2 fraction (fractions with retention times of 20 min or less), showed little antibacterial and antiviral activity. However, two fractions of *Lb. plantarum* LBP-K10 CF, F6 [*cis*-cyclo(L-Tyr-L-Pro), retention time: 9.5–10.5 min] and F7 [*cis*-cyclo(L-Val-L-Pro), retention time: 12.2–13.2 min], exhibited weak antibacterial and antifungal activity (Kwak et al., 2014). However, among the fractions tested, F13 [*cis*-cyclo(L-Leu-L-Pro)] and F17 [*cis*-cyclo(L-Phe-L-Pro)] exhibited the most significant and unique antibacterial, antifungal, and antiviral activities. Previous studies have shown that no other fraction of *Lb. plantarum* LBP-K10 CF exhibited antiviral activity, except for F13 and F17. Additionally, F17 showed a pronounced antifungal activity (Kwak et al., 2013; Kwak et al., 2018). The previous study also discovered a significant difference in antibiotic activity between fractions with HPLC chromatogram retention times of 20 min or less and those with retention times of 20 min or more in the case of *Ln. mesenteroides* LBP-K06 CF, similar to *Lb. plantarum* LBP-K10 (Liu et al., 2017). Even with the MeSPEf-45 fractionation, the quantities of the preceding fractions, excluding S8 and S11, were significantly low. The results suggest that only two fractions, S8 and S11, corresponding to F13 and F17, of the MeSPEf-45 fractions passed through the SPE cartridge retain antiviral activity. This study presents a sophisticated system that investigates the effect of the newly utilized MeSPE on the identification and recovery of biologically active CDPs, in comparison to the previously showcased AECs (Figure 8).

**TABLE 5:** The synergistic antiviral and antibacterial effects of the composite fractions comprising S8 and S11 against pathogenic microorganisms.

Type of pathogen	^*5*^ plaque number (±SD) non-MeSPEfc (CH_2_Cl_2_-extracted *Lb. plantarum* LBP-K10 CF)						
Influenza A virus (H3N2)							
^*1*^ References							
^*1*^ VC	109.5 ± 13.0						
^*1*^ UB	0						
^*1*^ WO	132.5 ± 13.60						

	Plaque Number (±SD) of MeSPEfs						
MeSPEf-45-derived combination	MeSPEf-45 S8	MeSPEf-45 S11		MeSPEf-45 S8 and S11	MeSPEf-45 without S8 and S11	MeSPEf-45	
Type of pathogen							
^*2*^ Influenza A virus (H3N2)	57.6 ± 9.58	54.0 ± 6.30		42.7 ± 5.45	^7^ N.S.	19.0 ± 5.30	

Type of pathogen	^*6*^ MIC (mg/L)						
	MeSPEf-45 S8	MeSPEf-45 S11	MeSPEf-45 S8 and S11		MeSPEf-45 without S8 and S11	MeSPEf-45	
Multidrug-resistant bacteria							
^*3*^ * S. aureus* 114,71^1^	13.75 ± 0.50	16.25 ± 2.50	10.75 ± 3.25		^8^ N.D.	7.50 ± 2.00	
^*4*^ * S.* Typhimurium 122,19^3^	14.25 ± 0.75	15.00 ± 2.25	10.35 ± 3.75		N.D.	8.99 ± 1.85

^a^ Reference, MDCK; VC, virus control; UB, unbound; WO, wash out, using non-MeSPEfc (CH_2_Cl_2_-extracted *Lb. plantarum* LBP-K10 CF).

^b^ Influenza A/H3N2 virus in this study were provided by the Korea National Institute of Health (KNIH).

Multidrug-resistant.

^c^ Gram-positive and.

^d^ Gram-negative bacteria supplied by the KNIH.

^e^ Data are presented as mean ± standard error of the mean from three independent experiments.

^f^ MIC: Minimum inhibitory concentration.

^g^ In this context, the term “non-significant” refers to two separate scenarios. The first scenario arises when the number of plaques acquired in the plaque assay exceeds the number of plaques in the virus control (VC). The second scenario occurs when the number of plaques is reduced, yet the variance is less than 10%.

^h^ In this context, the term “not detected” means that the MIC, values exceed 50 mg/mL.

As a representative in clinical trials involving CDPs with antiviral properties, spiro-DKP is a CDP derivative utilized to design chemokine receptors for selective C-C chemokine receptor type 5 (CCR5) antagonists, demonstrating promising antiviral effects (Habashita et al., 2006). CCR5 antagonists are powerful anti-HIV medications. There is increasing interest in CCR5 chemokine receptor antagonists as HIV-1 entry inhibitors for AIDS treatment. CCR5 is a G-protein-coupled receptor (GPCR), and the first antiviral CCR5 antagonist in this class was the spiro-DKP aplaviroc. The spiro-2,5-DKP template was chosen to design combinatorial libraries for targeting chemokine receptors to develop selective CCR5 antagonists. This template resembles spiropiperidine privileged structures found in various GPCR ligands. It is a fundamental heterocyclic scaffold that allows for diversity at up to four positions and can be synthesized in two steps from common α-amino acids using the Ugi reaction. The predicted 3D structure of spiro-DKP suggests that having three substituents on this template could align similarly to three side chains on the type I β-turn structure of the protein. The oral bioavailability of different aplaviroc formulations in rats and monkeys varied from 3% to 30%. It is 93% bound to human plasma, mainly metabolized by CYP3A4, and *in vitro* cell culture studies indicate it is a P-glycoprotein substrate (Adkison et al., 2005).

Certain CDPs are currently utilized as pharmaceutical agents, such as bicyclomycin and flinabulin (which are currently undergoing clinical trials), excluding 2,5-DKPs (Kohn and Widger, 2005; Blayney et al., 2020). Furthermore, cyclo(leucyl-glycine), a DKP compound designed as an analgesic and a therapeutic agent for Parkinson’s disease, has progressed into clinical trials (Bhargava, 1984; Chipkin and Latranyi, 1984). In contrast to the straightforwardly structured CDPs like cyclo(L-Phenylalanine-L-Proline), which have shown activity against 12 VRE bacterial strains and spiro-DKP, currently under investigation for its efficacy against the HIV virus, there has been a limited number of clinical trials conducted thus far utilizing the most basic structured CDPs, such as 2,5-DKP, a proline-containing CDP, for therapeutic purposes. Ampion represents a nearly unique case as a human serum albumin containing a small molecule compound. It has been formulated as the initial intra-articular injection intended for managing severe knee osteoarthritis. Ampion, also referred to as ‘aspartyl-alanyl diketopiperazine’ or ‘DA-DKP’, is an immunomodulatory molecule. DA-DKP has demonstrated efficacy in mitigating inflammation through the inhibition of pro-inflammatory cytokine production in T cells. The clinical trial NCT03349645, which focused on investigating DA-DKP, aimed to evaluate the safety of long-term treatment with Ampion for severe knee osteoarthritis (https://www.clinicaltrials.gov/study/NCT03349645?intr=NCT03349645&rank=1). However, the trial recently yielded unsuccessful results. Hence, it is postulated that the two categories of proline-containing CDPs delineated in this investigation could potentially yield clinical advantages provided that the diverse factors contributing to the shortcomings observed in the aspartyl-alanyl-diketopiperazine clinical trial mentioned earlier are rectified, leading to enhanced efficacy. The scope of applications for the two categories of CDPs outlined in this research is presently constrained to biopesticides, food additives, orthorexia, orthostasis, orthotics, and functional food materials aimed at mitigating foodborne illnesses.

## Conclusion

In this study, we aimed to introduce a more efficient method for quickly extracting large amounts of proline-containing CPDs, which are antiviral agents secreted extracellularly in *Lactobacillus* cultures. The high-throughput MeSPE technique accurately identifies CDPs by quantifying and comparing CDP fractions using CH_2_Cl_2_ extraction and a simple, reproducible HPLC fractionation method. The utilization of C18-SPE resin through the MeSPE method is more efficient in achieving a higher CDP yield and in eliminating organic acids and impurities from *Lb*. *plantarum* LBP-K10 CF. When compared to fractions obtained using the previous AEC method, which involved complex and multiple steps, the fractions with antiviral activity in MeSPEfs are easier to isolate CDPs. These CDPs were primarily eluted with 40% and 45% methanol. After HPLC fractionation, fractions from MeSPEf-20, MeSPEf-25, MeSPEf-30, and MeSPEf-35 exhibit moderate anti-influenza activity. MeSPEf-45 fractions S8 and S11 showed the highest activity against multidrug-resistant bacteria and influenza virus compared to other fractions of MeSPEfs. The anti-influenza activity in MeSPEfs fractions, like MeSPEf-40 and MeSPEf-45, is limited to the S8 and S11 fractions. Antiviral fractions S8 and S11 were identified as proline-based *cis*-cyclo(L-Leu-L-Pro) and *cis*-cyclo(L-Phe-L-Pro). We show that combining MeSPEf-45 fractions S8 and S11 with other fractions resulted in enhanced antibacterial and anti-influenza virus effects compared to using each fraction individually. This study strengthens our knowledge of managing and preventing viral infectious diseases using a specific CDP combination strategy and its applications. High-throughput MeSPE-derived MeSPEfs and their fractions offer innovative materials for selectively purifying significant quantities of potent antimicrobial CDPs from bacterial CF. Throughout the study, we present compelling evidence for our SPE technique to assess the impact of antiviral CDPs in complexes and identify specific bioactive CDPs.

## Data Availability

The original contributions presented in the study are included in the article/Supplementary Material; further inquiries can be directed to the corresponding author.

## Abbreviations

AEC, anion exchange chromatography; *cis*-cyclo(L-Leu-L-Pro), *cis*-cyclo(L-leucine-L-proline); *cis*-cyclo(L-Phe-L-Pro), *cis*-cyclo(L-phenylalanine-L-proline); CDPs, cyclic dipeptides; CH_2_Cl_2_, methylene chloride; CI, chemical ionization; DMEM, Dulbecco’s modified Eagle’s medium; EI, electron ionization; GC-MS, gas chromatography-mass spectrometry; prep-HPLC, preparative high-performance liquid chromatography; LAB, lactic acid bacteria; MDCK, Madin-Darby canine kidney; MeSPE, methanol solid-phase extraction; MeSPEf(s), methanol solid-phase extraction fraction(s) (eluate(s)); MIC, minimum inhibitory concentration; MRS, de Man, Rogosa and Sharpe; ODS, octadecyl silica; SPE, solid-phase extraction; TDW, triple-distilled water; *t*_*R*_, retention time.
