# Corrigendum: The high-throughput solid-phase extraction of *cis*-cyclo(L-Leu-L-Pro) and *cis*-cyclo(L-Phe-L-Pro) from *Lactobacillus plantarum* demonstrates efficacy against multidrug-resistant bacteria and influenza A (H3N2) virus

**DOI:** 10.3389/fmolb.2025.1605848

**Published:** 2025-04-15

**Authors:** Jaeyoung Son, Yeonju Hong, Hyeri Seong, Yoon Sin Oh, Min-Kyu Kwak

**Affiliations:** ^1^ Laboratory of Microbial Physiology and Biotechnology, Department of Food and Nutrition, Institute of Food and Nutrition Science, College of Bio-Convergence, Eulji University, Seongnam, Republic of Korea; ^2^ Department of Food and Nutrition, Institute of Food and Nutrition Science, College of Bio-Convergence, Eulji University, Seongnam, Republic of Korea

**Keywords:** cyclic dipeptides, cis-cyclo(L-Leu-L-Pro), cis-cyclo(L-Phe-L-Pro), solid-phase extraction, influenza A virus, *Lactobacillus plantarum* LBP-K10

In the published article, there was an error in the **Funding** statement. The name of one of the funders was omitted. The correct **Funding** statement appears below.

