# Supplementary material for: The high-throughput solid-phase extraction of cis-cyclo(L-Leu-L-Pro) and cis-cyclo(L-Phe-L-Pro) from Lactobacillus plantarum demonstrates efficacy against multidrug-resistant bacteria and influenza A (H3N2) virus
Source: Front Mol Biosci. 2024 May 17;11:1346598. doi: 10.3389/fmolb.2024.1346598 (PMC11140067; doi:10.3389/fmolb.2024.1346598)
Supplement: Supplementary file 1 [file Table1.pdf]

[Frontiers in Microbiology]
Manuscript ID: 1346598

Supplementary material:

The high-throughput solid-phase extraction of *cis*-cyclo(L-Leu-L-Pro) and *cis*-cyclo(L-Phe-L-Pro) from *Lactobacillus plantarum* demonstrates efficacy against multidrug-resistant bacteria and influenza A (H3N2) virus

Jaeyoung Son[a,1], Yeonju Hong[a,1], Hyeri Seong[a,1], Yoon Sin Oh[b], and Min-Kyu Kwak[a,]*

[a] Laboratory of Microbial Physiology and Biotechnology, Department of Food and Nutrition, Institute of Food and Nutrition Science, College of Bio-Convergence, Eulji University, Seongnam 13135, Republic of Korea

[b] Department of Food and Nutrition, Institute of Food and Nutrition Science, College of Bio-Convergence, Eulji University, Seongnam 13135, Republic of Korea

**** Correspondence:***
Min-Kyu Kwak, Laboratory of Microbial Physiology and Biotechnology, Department of Food and Nutrition, Institute of Food and Nutrition Science, College of Bio-Convergence, Eulji University, Seongnam 13135, Republic of Korea
Tel.: +82 31 740 7418; fax: +82 31 740 7370; E-mail: genie6@eulji.ac.kr (M.-K. Kwak).

Running title: *High-throughput screening of antiviral cyclic dipeptide combinations*

[1] These authors contributed equally to this work.

Keywords: cyclic dipeptides; *cis*-cyclo(L-Leu-L-Pro); *cis*-cyclo(L-Phe-L-Pro); solid-phase extraction; influenza A virus; *Lactobacillus plantarum* LBP-K10

Abbreviations: AEC, anion exchange chromatography; *cis*-cyclo(L-Leu-L-Pro), *cis*-cyclo(L-leucine-L-proline); *cis*-cyclo(L-Phe-L-Pro), *cis*-cyclo(L-phenylalanine-L-proline); CDPs, cyclic dipeptides; CH_2Cl_2, methylene chloride; CI, chemical ionization; DMEM, Dulbecco's modified Eagle's medium; EI, electron ionization; GC-MS, gas chromatography-mass spectrometry; prep-HPLC, preparative high-performance liquid chromatography; LAB, lactic acid bacteria; MDCK, Madin-Darby canine kidney; MeSPE, methanol solid-phase extraction; MeSPEf(s), methanol solid-phase extraction fraction(s) (eluate(s)); MIC, minimum inhibitory concentration; MRS, de Man, Rogosa and Sharpe; ODS, octadecyl silica; SPE, solid-phase extraction; TDW, triple-distilled water; t_R, retention time

SUPPLEMENTARY TABLES

SUPPLEMENTARY TABLE S1| Antagonism testing using the SPE samples obtained from LBP-K10 CFs against *B. subtilis* and *E. coli*.

MeOH %/[1]Antagonistic activity	Fraction no.										
	S1	S2	S3	S4	S5	S6	S7	S8	S9	S10	S11
	[2] 6.0–7.0	7.1–9.4	9.5–10.5	10.6–13.4	13.5–15.0	15.1–17.5	17.6–20.4	20.5–23.0	23.1–26.0	26.1–28.0	29.0–31.3
[a] 5%	–	–	–	–	–	–	–	–	–	–	–
[b] 5%	–	–	–	–	–	–	–	–	–	–	–
[a] 10%	–	–	–	–	–	–	–	–	–	–	–
[b] 10%	–	–	–	–	–	–	–	–	–	–	–
[a] 15%	–	–	–	–	–	–	–	–	–	+	+
[b] 15%	–	–	–	–	–	–	–	–	–	+	+
[a] 20%	–	–	–	–	–	–	–	+	–	+	++
[b] 20%	–	–	–	–	–	–	–	++	–	+	++
[a] 25%	–	–	–	–	–	–	–	++	–	+	++
[b] 25%	–	–	–	–	–	–	–	+	–	+	+
[a] 30%	–	–	–	–	–	–	–	+	–	+	++
[b] 30%	–	–	–	–	–	–	–	+	–	+	++
[a] 35%	–	–	–	–	–	–	–	+	–	–	++

[b] 35%	–	–	–	–	–	–	–	+	–	–	+
[a] 40%	–	–	–	–	–	–	–	++	–	–	+
[b] 40%	–	–	–	–	–	–	–	++	–	–	+
[a] 45%	–	–	–	–	–	–	+	+++	–	+	++
[b] 45%	–	–	–	–	–	–	+	++	–	–	+++
[a] 50%	–	–	–	–	–	–	+	–	–	–	–
[b] 50%	–	–	–	–	–	–	+	–	–	–	–

[1] Symbol: + indicates a size of less than 15 mm; ++ indicates a size of less than 22 mm; +++; indicates a size greater than 22 mm (Indicator strains: *B. subtilis* [a], *E. coli* [b])

[2] Retention Times (min).

* Data are presented as the mean ± standard error of the mean from three independent experiments.

SUPPLEMENTARY TABLE S2| The antibacterial activity of the SPE eluents at a methanol concentration of 45%.

Indicator strains	[1,2] MIC (mg/L)
Gram-positive bacteria	
Bacillus subtilis	7.14 ± 0.15
Staphylococcus aureus	9.57 ± 0.35
Streptococcus pneumoniae	10.01 ± 0.44
Gram-negative bacteria	
Salmonella Typhimurium	10.24 ± 1.11
Escherichia coli	9.525 ± 0.88
Shigella dysenterii	12.80 ± 0.97
Multidrug-resistant strain	
Gram-positive bacteria	
[3] *Staphylococcus aureus* 11471	8.15 ± 1.22
Gram-negative bacteria	
[4] *Salmonella* Typhimurium 12219 [c]	7.99 ± 0.71

[1] The values presented in the study denote the mean of three separate experiments with standard errors indicated as ±.

[2] MIC: Minimum inhibitory concentration.
Multidrug-resistant [3] Gram-positive and [4] Gram-negative bacteria were provided by the Korea National Institute of Health (KNIH).
